# Prognosis of Cryptogenic Stroke With Patent Foramen Ovale at Older Ages and Implications for Trials: A Population-Based Study and Systematic Review

**DOI:** 10.1001/jamaneurol.2020.1948

**Published:** 2020-07-06

**Authors:** Sara Mazzucco, Linxin Li, Peter M. Rothwell

**Affiliations:** 1 Wolfson Centre for Prevention of Stroke and Dementia, University of Oxford, Oxford, United Kingdom

## Abstract

**Importance:**

Patent foramen ovale (PFO) closure may prevent recurrent stroke after cryptogenic transient ischemic attack (TIA) or stroke (TIA/stroke) in patients aged 60 years or younger. Patent foramen ovale is associated with cryptogenic stroke in the older population, but risk of recurrence is unknown. Data on prognosis of patients receiving medical treatment at older ages (≥60 years) are essential to justify trials of PFO closure.

**Objective:**

To examine the age-specific risk of recurrence in patients with cryptogenic TIA/stroke with PFO.

**Design, Setting, and Participants:**

A prospective study was nested in the population-based Oxford Vascular Study between September 1, 2014, and March 31, 2019, with face-to-face follow-up for 5 years. A total of 416 consecutive patients with a diagnosis of cryptogenic TIA or nondisabling stroke, screened for PFO at a rapid-access TIA/stroke clinic, were included. A systematic review and meta-analysis of cohort studies reporting on ischemic stroke recurrence after cryptogenic TIA/stroke in patients with PFO who were receiving medical therapy alone, or with PFO vs no-PFO was conducted. Sample size calculation for future trials on PFO closure was performed for patients aged 60 years or older.

**Exposures:**

Patent foramen ovale and age as modifiers of risk of recurrent stroke after cryptogenic TIA/stroke in patients receiving only medical therapy.

**Main Outcomes and Measures:**

Risk of ischemic stroke recurrence in patients with cryptogenic TIA/stroke and PFO receiving medical therapy only, and in patients with vs without PFO, stratified by age (<65 vs ≥65 years), as well as sample-size calculation for future trials of PFO closure in patients aged 60 years or older.

**Results:**

Among the 153 Oxford Vascular Study patients with PFO (mean [SD] age, 66.7 [13.7] years; 80 [52.3%] men), recurrent ischemic stroke risk (2.05 per 100 patient-years) was similar to the pooled estimate from a systematic review of 23 other studies (9 trials and 14 observational studies) (2.00 per 100 patient-years; 95% CI, 1.55-2.58). However, there was heterogeneity between studies (*P* < .001 for heterogeneity), owing mainly to risk increasing with mean cohort age (meta-regression: *R*^2^ = 0.31; *P* = .003). In the pooled analysis of 4 studies including patients with or without PFO, increased risk of stroke recurrence with PFO was seen only at age 65 years or older (odds ratio, 2.5; 95% CI, 1.4-4.2; *P* = .001 for difference; *P* = .39 for heterogeneity). The pooled ischemic stroke risk was 3.27 per 100 patient-years (95% CI, 2.59-4.13) in 4 cohorts with mean age 60 years or older. Assuming the more conservative 2.0 per 100 patient-years ischemic stroke risk in the PFO nonclosure arms of future trials in patients aged 60 years or older, projected sample sizes were 1080 per arm for 80% power to detect a 33% relative risk reduction.

**Conclusions and Relevance:**

The findings of this study suggest that age is a determinant of risk of ischemic stroke after cryptogenic TIA/stroke in patients with PFO, such that trials of PFO closure at older ages are justified; however, projected sample sizes are large.

## Key Points

**Question:**

Is the prognosis of cryptogenic transient ischemic attack and stroke in patients receiving medical treatment alone associated with the presence of patent foramen ovale and age?

**Findings:**

In this population-based study, risk of recurrent ischemic stroke (2.05 per 100 patient-years) was consistent with the pooled estimate from a systematic review and meta-analysis (2 per 100 patient-years). Risk increased with mean cohort age, and only in patients with cryptogenic transient ischemic attack and stroke aged 65 years or older was patent foramen ovale vs no patent foramen ovale associated with a higher risk of recurrent ischemic stroke.

**Meaning:**

The findings of this study suggest that a higher risk of stroke recurrence at older ages in patients with patent foramen ovale warrants trials of patent foramen ovale closure in older patients.

## Teaser

This systematic review and meta-analysis examines the recurrence of ischemic stroke and possible use of patent foramen ovale closure in patients aged 60 years or older.

## Introduction

The causal role of patent foramen ovale (PFO) in patients with cryptogenic transient ischemic attack (TIA) and/or stroke (TIA/stroke) has been reinforced by randomized clinical trials showing that percutaneous closure reduces the risk of recurrent stroke, mostly in patients aged 60 years or younger.^1,2,3,4,5^ However, older patients account for most strokes and PFO is associated with cryptogenic TIA/stroke in patients at older ages,^6,7^ with a substantial burden at a population level (approximately 6000 patients aged >60 years with large PFO and cryptogenic TIA/nondisabling stroke every year in the UK^6^). To our knowledge, there is no evidence from randomized clinical trials that PFO closure is effective in secondary prevention of stroke in patients older than 60 years.

In published trials of PFO closure, the risk of stroke recurrence is low even in the nonclosure arm (1.27 per 100 person-years),^8^ but this risk might be higher at older ages.^9^ However, PFO closure might be less effective and/or higher risk in older patients than in younger ones; therefore, new trials of PFO closure at older ages are required. Data on age-specific prognosis on medical treatment alone are warranted to justify trials of PFO closure at older ages and inform design of the trials.

We examined the age-related risk of ischemic stroke recurrence in a large, population-based cohort after cryptogenic TIA/nondisabling stroke in patients with PFO. We also performed a systematic review of cohort studies reporting ischemic stroke recurrence after cryptogenic TIA/stroke in patients with PFO receiving medical therapy alone or with PFO vs no PFO and pooled our data in a meta-analysis stratified by age, as well as in a meta-regression. We then estimated the sample size for future trials of PFO closure vs medical treatment alone in secondary prevention of cryptogenic TIA/stroke in patients with PFO aged 60 years or older.

## Methods

### OxVasc Study

This study was nested in the Oxford Vascular (OxVasc) Study, an ongoing, population-based study of the incidence and outcome of all acute vascular events in a population of 92 728 individuals, irrespective of age, registered with 100 primary care physicians in 9 practices in Oxfordshire, UK. Multiple methods of ascertainment are used for patients with TIA/stroke, as detailed elsewhere^10^ and summarized in the eMethods in the Supplement, including a daily, rapid-access TIA/stroke clinic to which participating physicians and the local emergency department refer individuals with suspected TIA or nondisabling stroke. As part of the OxVasc Phenotyped Cohort, consecutive, eligible patients attending this clinic with an acute event or for 3-month follow-up after an inpatient admission were screened for PFO between September 1, 2014, and March 31, 2019, and followed up until December 1, 2019. The OxVasc study and TCD assessment were approved by the local ethics committee and written informed consent was obtained from all participants, or assent was obtained from relatives in the case of cognitive impairment or speech difficulty.

Patients were assessed by a neurologist or stroke physician, and all presentations and investigations were reviewed by the senior study neurologist (P.M.R.). Demographic data, history of atrial fibrillation, atherosclerotic risk factors (ie, male sex, as well as history of hypertension, diabetes, smoking, hypercholesterolemia, and coronary or peripheral vascular disease) were recorded during face-to-face interviews and cross-referenced with primary care medical records.^11^ Patients routinely had 12-lead electrocardiography (ECG) and routine blood tests (ie, complete blood cell count, clotting profile, C-reactive protein, erythrocyte sedimentation rate, liver function, kidney function, thyroid function, electrolytes, and lipid profile) after the event. All patients underwent magnetic resonance brain and vascular imaging if not contraindicated (3-T magnetic resonance imaging with time-of-flight magnetic resonance angiography of the intracranial vessels and contrast-enhanced magnetic resonance angiography of the large neck arteries), or brain computed tomographic imaging with contrast-enhanced angiography or duplex ultrasonography if magnetic resonance imaging was contraindicated. Patients with cryptogenic TIA or stroke or those younger than 55 years also had thrombophilia screening, vasculitis screening, and genetic testing when appropriate. Clinical workup was completed with 5-day ambulatory ECG recording (R-test) and transthoracic echocardiography.^11^

Contrast-enhanced transcranial Doppler (TCD) (bubble-TCD) sonography (Doppler Box; Compumedics DWL) was performed by 1 of 2 experienced operators (S.M. and L.L.) who were blind to the patient’s clinical presentation. As a contrast agent, agitated saline was used in all cases according to accepted guidelines.^12^ A large PFO was defined as a shunt with 20 or more microbubbles recorded. Since November 15, 2015, if a temporal bone window was not suitable for monitoring, the basilar artery was monitored through a transoccipital approach.^13^ Designation of PFO status was made at the time of assessment, and recordings were archived.

The OxVasc definition for stroke and TIA is given in the eMethods in the Supplement. Cause of ischemic events was classified according to the Trial of Org 10172 in Acute Stroke Treatment criteria.^14^ Events were classified as cryptogenic if the diagnostic workup included at least brain imaging, ECG, and complete vascular imaging, and no clear cause was found. Patent foramen ovale alone was not considered as a criterion for cardioembolic stroke.^11^

To identify any recurrent stroke, patients were followed up face-to-face or via telephone if they had moved out of the study area at 1, 3, 6, 12, 24, and 60 months. All recurrences during follow-up would be identified through additional sources, such as daily case ascertainment (hot pursuit as described in the eMethods in the Supplement) and review of primary care records. Deaths with underlying causes during follow-up were recorded by direct follow-up via primary care records and by centralized registration with the Office for National Statistics.

### Systematic Review

We carried out a systematic review according to the Preferred Reporting Items for Systematic Reviews and Meta-analyses (PRISMA) reporting guideline for meta-analyses.^15^ We searched MEDLINE and Web of Science for articles published from inception until March 31, 2020, using the terms *stroke*, *cryptogenic stroke*, *stroke of undetermined aetiology, embolic stroke of undetermined source, foramen ovale, PFO*, *atrial septal abnormality*, *interatrial septal abnormality*, *right-to-left shunt*, *prognosis*, and *recurrent stroke*. Full search strategy details are given in the eMethods in the Supplement).

We restricted the search to cohorts with more than 100 patients with cryptogenic TIA/stroke, reporting on stroke recurrence in patients with PFO who were receiving medical therapy alone. We did not limit the search to English-language studies. We also hand-searched reference lists of all articles identified, of the publications related to the component databases of the Risk of Paradoxical Embolism Study,^16^ and those of any previous systematic reviews. We contacted study authors to retrieve missing data as appropriate.

Eligible studies included case-control, cohort, population-based studies or randomized clinical trials reporting on recurrent ischemic stroke after cryptogenic events in patients with PFO who were receiving medical treatment alone. Two of us (S.M. and L.L.) independently screened the references and identified eligible studies. Any discrepancy was solved via discussion or involving another one of us (P.M.R.). In case of duplicate studies, we included only the report with the most informative and complete data.

Two of us (S.M. and L.L.) independently extracted data from eligible articles using a standardized data extraction form. Information obtained included study type, setting of enrolment, years of enrolment, number of patients with cryptogenic stroke vs stroke of known causes, number of patients with cryptogenic stroke with vs without PFO, age cutoff and mean age, follow-up duration, type of index event, stroke subtype classification (Trial of Org10172 in Acute Stroke Treatment^14^ vs other); assessment for index event (complete, incomplete, or unreported), PFO screening modality (transthoracic echocardiography, transesophageal echocardiography, or bubble-TCD), prolonged ECG monitoring, baseline modified Rankin scale, medical treatment after index event (antiplatelet agents or anticoagulation), intracranial hemorrhages, assessment for recurrent event (direct, indirect, or unreported), type of recurrent event, and number of patients with ischemic stroke recurrences with vs without PFO.

The definition of PFO was based on the demonstration of right-to-left shunt both for studies using TCD^12^ or transthoracic or transesophageal echocardiography,^17^ the definition of right-to-left shunt size in the latter examination usually being based on the maximum number of microbubbles reported in the left atrium during the first 3 cardiac cycles after detection in the right atrium; a large right-to-left shunt usually comprised more than 20 to 25 microbubbles.^2,4^ Assessment of the index event was considered complete if the diagnostic workup included at least brain imaging, ECG, and extracranial imaging.^11^ Face-to-face assessment with study physicians in the acute phase of a possible recurrent event was considered as direct assessment for recurrent events. To assess study quality, we used the Newcastle-Ottawa Scale.^18^

### Outcomes

First, we aimed to estimate the absolute risk of ischemic stroke recurrence (or ischemic and hemorrhagic stroke when ischemic only was not reported) per 100 patient-years in patients with cryptogenic TIA/stroke and PFO receiving medical therapy only, and its association with mean study age, both in the OxVasc Study cohort and in cohorts from the systematic review. Second, we aimed to determine any excess risk of recurrent stroke in patients with vs without PFO, stratified by age (<65 or ≥65 years), by pooling OxVasc data with data from the systematic review. Third, we aimed to calculate the desirable sample size for future trials of PFO closure and medical treatment after cryptogenic stroke/TIA in patients aged 60 years or older.

### Statistical Analysis

Absolute risk of ischemic stroke recurrence per 100 patient-years was calculated in the OxVasc Study cohort and for each of the included studies in the systematic review (or ischemic and hemorrhagic stroke when ischemic only was not reported) on the basis of the data provided in the main article, the supplemental material, or by the author on request. Pooled estimates of absolute risk were obtained by meta-analysis with the 95% CI of the pooled risk estimate calculated to allow for extrabinomial variation,^19^ to avoid artificially narrow intervals produced by standard methods of calculating 95% CI in case of heterogeneity between studies. To test for age as a potential source of between-study heterogeneity, we regressed the ischemic stroke recurrence rate against study mean age, weighted by the inverse variance of risk estimate.^20^

In the OxVasc Study cohort and in studies that reported on both groups, meta-analysis with Mantel-Haenszel-Peto methods was used to determine excess risk of recurrent stroke in patients with PFO vs those without PFO by calculating the odds ratio (OR) with 95% CI for ischemic stroke recurrence in patients with cryptogenic events and PFO vs patients without PFO stratified by age (<65 or ≥65 years).

We estimated the projected sample size for trials of PFO closure vs medical treatment only after cryptogenic TIA/stroke in patients aged 60 years or older with 80% power to detect either a 66% risk reduction for the interventional closure arm, which was expected on the basis of trials predominantly on antiplatelet treatment in the younger population, or a more conservative 33% reduction to allow for the higher background risk of stroke in the older population or a comparison with anticoagulation.^21^

## Results

Of 456 consecutive patients in the OxVasc Study with a diagnosis of cryptogenic TIA/nondisabling stroke, bubble-TCD was obtained in 416 patients, with intolerance to supine position, lack of bone window, and cannulation issues being the most common reasons for lack of scanning. Of the 416 scanned patients, 153 had a positive bubble-TCD (36.78%, mean [SD] age, 66.7 [13.7] years). Characteristics of patients with cryptogenic TIA/nondisabling stroke with and without PFO, stratified by age (<65 or ≥65), are reported in the Table.

**Table.** Baseline Characteristics of Cryptogenic Events in the Oxford Vascular Study Cohort, Stratified by Age

Characteristic	All Patients			≥65 y		
	No. (%)		*P* value	No. (%)		*P* value
	PFO (n = 153)	No PFO (n = 263)		PFO (n = 98)	No PFO (n = 180)	
Age, mean (SD), y	66.7 (13.7)	69.3 (12.7)	.06	75.1 (6.4)	76.4 (6.6)	.09
Male sex	80 (52.3)	126 (47.9)	.39	47 (48.0)	78 (43.3)	.46
Index event						
Transient ischemic attack	110 (71.9)	193 (73.4)	.74	45 (79.6)	135 (75.0)	.39
Ischemic stroke	43 (28.1)	70 (26.6)		20 (20.4)	45 (25.0)	
Previous vascular event						
Myocardial infarction	6 (3.9)	9 (3.4)	.79	5 (5.1)	7 (3.9)	.63
Peripheral vascular disease	3 (2.0)	5 (1.9)	.97	3 (3.1)	1 (1.7)	.45
Transient ischemic attack	14 (9.2)	15 (5.7)	.18	12 (12.2)	14 (7.8)	.22
Stroke	12 (7.8)	22 (8.4)	.85	7 (7.1)	17 (9.4)	.51
Known vascular risk factors						
Hypertension	72 (47.1)	152 (57.8)	.03	58 (59.2)	122 (67.8)	.15
Diabetes	22 (14.4)	34 (12.9)	.68	14 (14.3)	27 (15.0)	.87
Hyperlipidemia	61 (39.9)	91 (34.6)	.28	49 (50.0)	71 (39.4)	.09
Valvular heart disease	5 (3.3)	11 (4.2)	.64	3 (3.1)	8 (4.4)	.57
Cardiac failure	3 (2.0)	2 (0.8)	.28	3 (3.1)	2 (1.1)	.24
Venous thrombosis	4 (2.6)	9 (3.4)	.65	3 (3.1)	7 (3.9)	.72
History of smoking	87 (56.9)	130 (49.4)	.14	52 (53.1)	84 (46.7)	.31
PFO size						
Small (shunt with <20 microbubbles)	92 (60.1)	NA	NA	63 (64.3)	NA	NA
Medium-large (shunt with ≥20 microbubbles)	61 (39.9)	NA	NA	35 (35.7)	NA	NA
Medication at discharge						
Warfarin alone	0 (0)	2 (0.8)	.54	0 (0)	1 (0.6)	.73
DOACs alone	0 (0)	1 (0.4)		0 (0)	0 (0)	
Antiplatelet	152 (99.3)	257 (97.7)		97 (99.0)	177 (98.3)	
Antiplatelet plus warfarin	1 (0.4)	1 (0.7)		1 (1.0)	1 (0.6)	
Antiplatelet plus DOACs	0 (0)	2 (0.8)		0 (0)	1 (0.6)	

Abbreviations: DOACs, direct oral anticoagulants; NA, not applicable; PFO, patent foramen ovale.

Of 577 potentially eligible records identified for the systematic review, we identified 28 eligible articles reporting on 23 studies^2,3,4,5,9,17,22,23,24,25,26,27,28,29,30,31,32,33,34,35,36,37,38^ (9 trials and 14 observational studies) including 4889 patients with cryptogenic TIA/stroke and PFO who were receiving medical treatment alone (eFigure 1 in the Supplement). Noneligible studies included observational studies^39,40,41,42,43,44^ and 1 randomized clinical trial (47 patients with cryptogenic stroke/TIA randomized to either warfarin or aspirin)^45^ with a population below the threshold for inclusion (<100 patients with cryptogenic TIA/stroke enrolled); observational studies on an unsuitable population (mostly retrospective studies selecting patients on the basis of echocardiographic finding of a PFO, where the analysis included patients without stroke, or patients with both cryptogenic and noncryptogenic TIA/stroke)^46,47,48,49,50,51,52,53,54^; secondary publications of an included study^55,56^; and a study that reported only composite outcomes of TIA/stroke and peripheral embolism.^57^ We contacted 8 authors and obtained unpublished data from 4 individuals.^9,22,23,24,58^ Study characteristics are detailed in the eResults and eTable 1 in the Supplement). The quality assessment of the studies is reported in eTable 2 in the Supplement.

**Figure 1.**
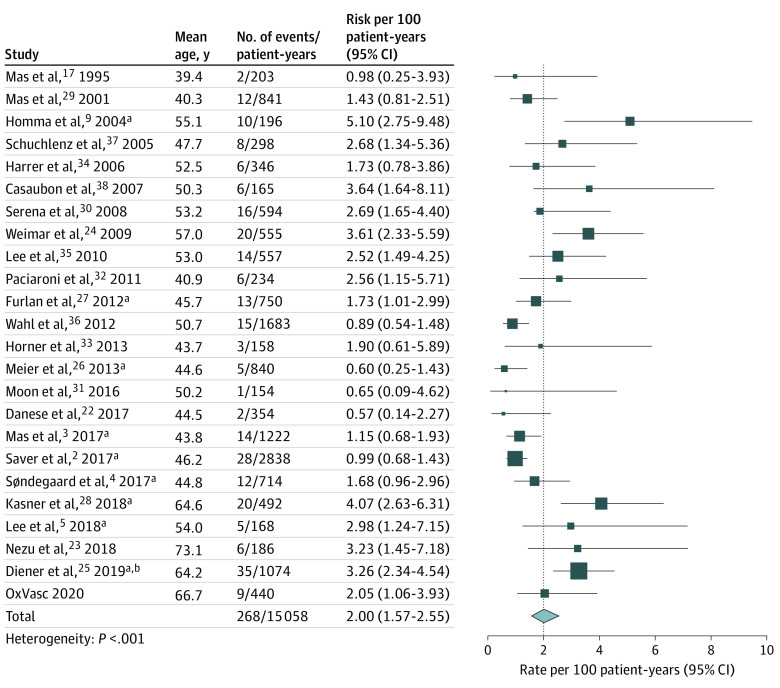
Absolute Risk of Recurrent Ischemic Stroke in Patients With Patent Foramen Ovale Receiving Medical Treatment Alone OxVasc indicates Oxford Vascular Study. ^a^Randomized trials. ^b^All recurrent strokes (ie, not recurrent ischemic strokes only).

In OxVasc patients with cryptogenic TIA/stroke and PFO, a total of 9 recurrent ischemic strokes occurred during 440 patient-years, with an absolute risk of 2.05 per 100 patient-years (Figure 1). Of these, 5 patients per 329 patient-years had TIA as an index event (risk, 1.5 per 100 patient-years) and 4 per 111 patient-years had a stroke (risk, 3.6 per 100 patient-years). On pooling with the 23 other eligible studies from the systematic review, risk per 100 patient-years of ischemic stroke was 2.00 (95% CI, 1.57-2.55) (Figure 1), based on 268 recurrent events during 362 324 patient-years. One included study with patient mean age of 64.2 years reported the risk of all strokes rather than ischemic strokes only,^25^ contributing to the analysis with 6 of 268 events overall. A sensitivity analysis excluding this study is shown in eFigure 2 in the Supplement.

**Figure 2.**
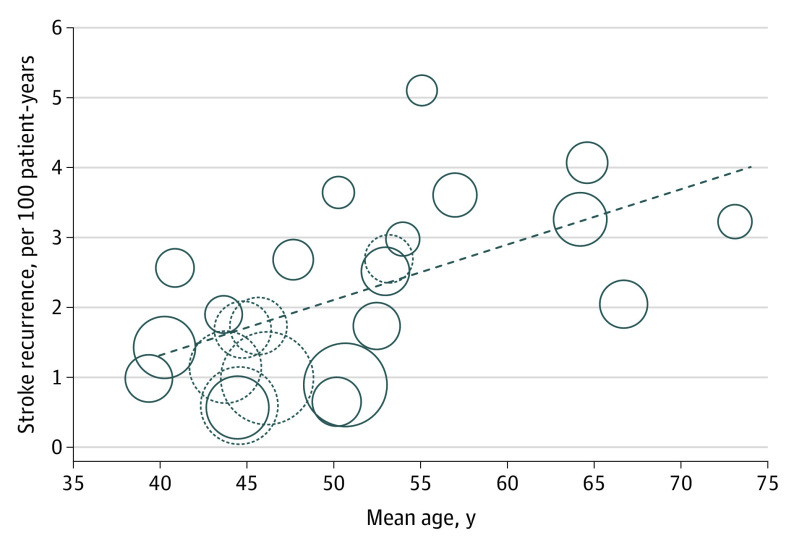
Meta-Regression Analysis Between Recurrent Ischemic Stroke Risk and Mean Study Age Dotted circles represent the medical arm of patent foramen ovale closure trials.

There was substantial heterogeneity between studies (*P* < .001 for heterogeneity) in the risk of ischemic stroke, which was partly explained by higher risk with increasing mean age of the study cohort (meta-regression: *R*^2^ = 0.31; *P* = .003), with absolute risk increasing by about 50% in relative terms for every 10 years of age (Figure 2). The pooled ischemic stroke risk at age 60 years or older was 3.27 per 100 patient-years (95% CI, 2.59-4.13).

Pooled recurrent ischemic stroke risk was 1.26 per 100 patient-years (95% CI, 0.91-1.75) among the randomized clinical trials of PFO closure (mean [SD] age, 46.51 [9.55] years),^2,3,4,5,26,27^ and 2.37 per 100 patient-years (95% CI, 1.84-3.05) for the other cohorts (mean [SD] age, 51.79 [11.37]), including the 3 randomized clinical trials of antithrombotic treatment.^9,25,28^ Excluding these 3 trials, the pooled risk was 2.04 per 100 patient-years (95% CI, 1.55-2.69) for the observational cohorts only.^17,22,23,24,29,30,31,32,33,34,35,36,37,38^

**Figure 3.**
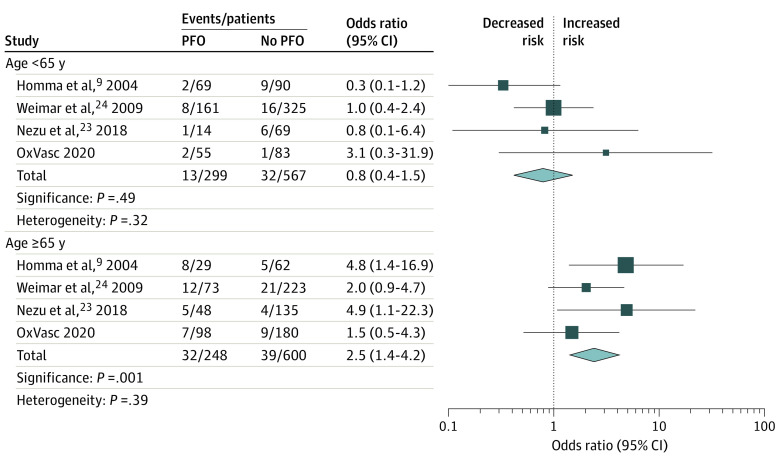
Risk of Ischemic Stroke Recurrence After Cryptogenic Transient Ischemic Attack/Stroke in Patients With Patent Foramen Ovale (PFO) vs Patients Without PFO OxVasc indicates Oxford Vascular Study.

In 7 of the above-mentioned studies, ischemic stroke risk was reported in patients with cryptogenic TIA/stroke with PFO vs those without PFO.^9,17,23,24,28,29,30^ When pooling the subgroup of studies reporting this association stratified by age^9,23,24^ with the OxVasc Study data, we found that the association between PFO and stroke risk increased with age (<65 years: OR, 0.8; 95% CI, 0.4-1.5; ≥65 years: OR, 2.5; 95% CI, 1.4-4.2; *P* = .001 for difference; *P* = .39 for heterogeneity) (Figure 3).

Although the pooled ischemic stroke risk per 100 patient-years at age 60 years or older was 3.27 per 100 patient-years (95% CI, 2.59-4.13), for the purpose of sample-size calculation, we assumed a more conservative estimate for the nonclosure arms of future trial cohorts of 2.0 per 100 patient-years. The PFO closure trials showed 66% risk reductions in the interventional arm compared with medical treatment only; however, we ran the main analysis based on a hypothetical 33% risk reduction, which would take into consideration increased background risk of stroke due to other causes in the population aged 60 years or older, or a comparison with anticoagulation. For trials of PFO closure vs medical treatment, projected total trial sample size was 2160 (1080 per arm) with 33% risk reduction and 432 (216 per arm) with 66% risk reduction for trials of PFO closure vs medical treatment (eTable 3 in the Supplement).

## Discussion

Our data suggest that age is a determinant of prognosis after cryptogenic TIA/stroke in patients with PFO who are receiving medical treatment alone. Reasons for this age-related increase in risk of recurrent stroke associated with PFO might include the increasing prevalence of venous thrombosis with age,^59^ increasing pulmonary pathologic changes and right-ventricular pressure potentially uncovering latent or previously nonsignificant right-to-left shunts,^11^ and increase in PFO size with age, albeit that PFO prevalence appears to decrease.^60^ However, it cannot necessarily be assumed that the proportion of strokes that are causally related to PFO is maintained at older ages, as the prevalence of other causes of stroke will tend to increase with age. Nevertheless, we also found that the relative risk of ischemic stroke in patients with cryptogenic TIA/stroke with vs without PFO was greatest at older ages.

Our systematic review included cohorts from both randomized and nonrandomized studies that included patients with cryptogenic TIA/stroke with PFO who were receiving only medical treatment. We found significant heterogeneity between studies in terms of absolute risk of recurrent stroke, which was mainly explained by the age of the patients. In addition, PFO closure trials^2,3,4,5,26,27^ suggested a lower absolute risk of stroke recurrence during medical treatment only (1.26 per 100 patient-years in our meta-analysis), while observational cohorts and nonclosure trials suggested a higher risk of 2.37. However, this difference was also mainly associated with the difference in mean age between cohorts rather than by the difference in the nature of the study, with mean age being significantly lower among closure trials (46.51 vs 51.79 years). Studies in which the mean age of the patients was 60 years or older included large randomized clinical trials, such as NAVIGATE ESUS^28^ and RE-SPECT ESUS,^25^ with overall mean ischemic stroke risk per 100 patient-years as high as 3.27—almost 3 times higher than the risk in PFO closure trials.

By comparing the risk of stroke recurrence in patients with vs without PFO stratified by age, we noted an apparent age-related excess risk of recurrent stroke conferred by PFO, with stroke risk associated with PFO being greater only in patients with PFO who were 65 years or older. This finding suggests that the increase in stroke risk with age in our main analysis of PFO cohorts is not simply due to the increase in the background vascular risk burden with age.^21^ The choice of a different age threshold of 65 years (as opposed to 60 years used in PFO trials) for our analysis of risk for PFO vs no PFO was related to the age thresholds reported in the studies included in the analysis.^9,23,24^

Although randomized clinical trials of PFO closure showed benefit in younger patients, we did not find an increased risk of stroke recurrence in younger patients with PFO. However, these findings are not inconsistent, but instead suggest that PFO-associated cryptogenic stroke in younger patients has a relatively low risk of recurrence, albeit lowered further by closure. It is also possible that increasingly effective medical treatment in more recent studies might be associated with reduced risks. Given the small numbers of studies and outcome events in this analysis, it is also possible that our study lacked statistical power to detect potentially clinically important associations reliably.

Older patients account for most strokes, and a previous study suggested that cryptogenic TIA/stroke associated with PFO at older ages is common,^6^ with an estimated number of 6000 cryptogenic TIA/nondisabling strokes every year in the UK in patients older than 60 years with large PFOs, suggesting that recruitment into future randomized clinical trials of PFO closure in the older population might be easier than it was for trials in the younger population. However, in our sample size calculation for possible future trials of PFO closure in the older population, we included a scenario in which the relative reduction in stroke risk with closure was smaller than reported in the previous trials in younger patients (33% risk reduction vs 66% reported for the younger population). In the older population, the increased background risk of stroke due to other causes, such as atrial fibrillation, could attenuate the benefit of PFO closure, as might preexisting anticoagulation for other conditions. Moreover, trials in the older population might show that PFO closure is higher risk than in younger patients,^61^ for example, owing to the potentially higher risk of procedure-related atrial fibrillation. However, similar concerns about the outcomes of other procedures at older ages have proved to be unfounded in the past.^62,63^ Either way, off-label closure outside clinical trials should not be routinely advocated for older patients.

### Strengths and Limitations

Our study has a number of strengths. It included a large, population-based evaluation of the prognosis of patients with cryptogenic TIA/stroke and PFO who were receiving only medical treatment and a meta-analysis of all available data from trial and nontrial cohorts. However, our study has some limitations. First, we cannot rule out a bias in some of the studies included in the analysis, which could produce an artificially low risk of recurrent stroke in medically treated patients. It is clear in some of the observational studies^22,31,32,33,37,38^ that patients considered at high risk of recurrent stroke (presence of atrial septal aneurysm, large shunt, multiple index events) underwent percutaneous closure of PFO and were not therefore included in the study of prognosis while receiving only medical treatment. This bias might be true even for PFO closure trials, which had slow enrollment owing to the wide offer of off-label PFO closure to patients.^64^ It is therefore possible that our meta-analysis for patients receiving medical treatment underestimated the absolute risk of stroke. Moreover, because such inclusion bias might be greater in younger patients, it is possible that we may have overestimated the association between stroke risk and age. Second, we found a limited number of studies including older patients, so our meta-regression was based on fewer studies at older ages. However, some of these studies were large and contributed a significant number of patients and events.^25,28^ Third, assuming a 10% risk of recurrence over 5 years based on a rate of 2 per 100 patient-years could lead to an underestimated sample size calculation, as most of the included studies for the risk calculation did not reach 5 years of follow-up. Fourth, OxVasc Study participants with right-to-left shunt on bubble-TCD did not systematically undergo transesophageal echocardiography because of the absence of evidence on the benefit of PFO closure at the time of our study. Potentially, a small proportion of the right-to-left shunts found could be due to non-PFO sources, usually pulmonary shunts; however, most TCD-detected right-to-left shunts are shown to be due to PFO on transesophageal echocardiography.^65^ Fifth, owing to the small number of recurrent events, the OxVasc Study did not have sufficient power to reliably determine whether the recurrence risk was greater after stroke than after TIA. Sixth, the data from the OxVasc Study may not be fully generalizable to routine clinical practice in all centers. In the OxVasc Study, patients are extensively investigated through prolonged 5-day ECG monitoring to detect occult paroxysmal atrial fibrillation, transthoracic echocardiography, and home telemetric blood pressure monitoring, and are treated equally aggressively with dual antiplatelet therapy at least for 1 month, followed by single antiplatelet therapy thereafter, and intensive therapy to lower blood pressure and cholesterol levels.

## Conclusions

Increasing age is associated with a higher risk of ischemic stroke after cryptogenic TIA/stroke in patients with PFO. The findings of this study suggest that trials of PFO closure in secondary prevention of cryptogenic TIA/stroke at older ages are justified, although sample sizes would need to be large if the relative reduction in risk of recurrent stroke with closure is smaller than in previous trials of younger patients.

## Notes

Supplement. eMethods. OxVasc Methodology eResults. Characteristics of Studies Included in the Systematic Review eFigure 1. PRISMA Flow Chart eTable 1. Data Extraction Forms With Included Papers eTable 2. Quality Assessment for Included Studies With the Newcastle-Ottawa Scale eFigure 2. Subgroup Analysis on the Absolute Risk of Recurrent Ischaemic Stroke Calculated on the 23 Studies Reporting This Outcome eTable 3. Sample Size Calculation for Trials of PFO Closure Versus Medical Treatment Only After Cryptogenic TIA/Stroke in Patients ≥60 Years of Age

## References

[1] Carroll JD, Saver JL, Thaler DE, ; RESPECT Investigators Closure of patent foramen ovale versus medical therapy after cryptogenic stroke. N Engl J Med. 2013;368(12):1092-1100. doi:10.1056/NEJMoa1301440 23514286

[2] Saver JL, Carroll JD, Thaler DE, ; RESPECT Investigators Long-term outcomes of patent foramen ovale closure or medical therapy after stroke. N Engl J Med. 2017;377(11):1022-1032. doi:10.1056/NEJMoa1610057 28902590

[3] Mas JL, Derumeaux G, Guillon B, ; CLOSE Investigators Patent foramen ovale closure or anticoagulation vs antiplatelets after stroke. N Engl J Med. 2017;377(11):1011-1021. doi:10.1056/NEJMoa1705915 28902593

[4] Søndergaard L, Kasner SE, Rhodes JF, ; Gore REDUCE Clinical Study Investigators Patent foramen ovale closure or antiplatelet therapy for cryptogenic stroke. N Engl J Med. 2017;377(11):1033-1042. doi:10.1056/NEJMoa1707404 28902580

[5] Lee PH, Song JK, Kim JS, Cryptogenic stroke and high-risk patent foramen ovale: the DEFENSE-PFO Trial. J Am Coll Cardiol. 2018;71(20):2335-2342. doi:10.1016/j.jacc.2018.02.046 29544871

[6] Mazzucco S, Li L, Binney L, Rothwell PM Prevalence of patent foramen ovale in cryptogenic transient ischaemic attack and non-disabling stroke at older ages: a population-based study, systematic review, and meta-analysis. Lancet Neurol. 2018;17(7):609-617. doi:10.1016/S1474-4422(18)30167-4 29887162 PMC6004554

[7] Handke M, Harloff A, Olschewski M, Hetzel A, Geibel A Patent foramen ovale and cryptogenic stroke in older patients. N Engl J Med. 2007;357(22):2262-2268. doi:10.1056/NEJMoa071422 18046029

[8] Turc G, Calvet D, Guérin P, Sroussi M, Chatellier G, Mas JL; CLOSE Investigators Closure, anticoagulation, or antiplatelet therapy for cryptogenic stroke with patent foramen ovale: systematic review of randomized trials, sequential meta-analysis, and new insights from the CLOSE Study. J Am Heart Assoc. 2018;7(12):e008356. doi:10.1161/JAHA.117.008356 29910193 PMC6220551

[9] Homma S, DiTullio MR, Sacco RL, Sciacca RR, Mohr JP; PICSS Investigators Age as a determinant of adverse events in medically treated cryptogenic stroke patients with patent foramen ovale. Stroke. 2004;35(9):2145-2149. doi:10.1161/01.STR.0000135773.24116.18 15232117

[10] Rothwell PM, Coull AJ, Giles MF, ; Oxford Vascular Study Change in stroke incidence, mortality, case-fatality, severity, and risk factors in Oxfordshire, UK from 1981 to 2004 (Oxford Vascular Study). Lancet. 2004;363(9425):1925-1933. doi:10.1016/S0140-6736(04)16405-2 15194251

[11] Li L, Yiin GS, Geraghty OC, ; Oxford Vascular Study Incidence, outcome, risk factors, and long-term prognosis of cryptogenic transient ischaemic attack and ischaemic stroke: a population-based study. Lancet Neurol. 2015;14(9):903-913. doi:10.1016/S1474-4422(15)00132-5 26227434 PMC5714616

[12] Jauss M, Zanette E Detection of right-to-left shunt with ultrasound contrast agent and transcranial Doppler sonography. Cerebrovasc Dis. 2000;10(6):490-496. doi:10.1159/000016119 11070388

[13] Del Sette M, Dinia L, Rizzi D, Sugo A, Albano B, Gandolfo C Diagnosis of right-to-left shunt with transcranial Doppler and vertebrobasilar recording. Stroke. 2007;38(8):2254-2256. doi:10.1161/STROKEAHA.106.479485 17600238

[14] Adams HP Jr, Bendixen BH, Kappelle LJ, Classification of subtype of acute ischemic stroke: definitions for use in a multicenter clinical trial: TOAST, Trial of Org 10172 in Acute Stroke Treatment. Stroke. 1993;24(1):35-41. doi:10.1161/01.STR.24.1.35 7678184

[15] Liberati A, Altman DG, Tetzlaff J, The PRISMA statement for reporting systematic reviews and meta-analyses of studies that evaluate healthcare interventions: explanation and elaboration. BMJ. 2009;339:b2700. doi:10.1136/bmj.b2700 19622552 PMC2714672

[16] Thaler DE, Di Angelantonio E, Di Tullio MR, The Risk of Paradoxical Embolism (RoPE) Study: initial description of the completed database. Int J Stroke. 2013;8(8):612-619. doi:10.1111/j.1747-4949.2012.00843.x 22883936 PMC4060865

[17] Mas JL, Zuber M; French Study Group on Patent Foramen Ovale and Atrial Septal Aneurysm Recurrent cerebrovascular events in patients with patent foramen ovale, atrial septal aneurysm, or both and cryptogenic stroke or transient ischemic attack. Am Heart J. 1995;130(5):1083-1088. doi:10.1016/0002-8703(95)90212-0 7484740

[18] Luchini C, Stubbs B, Solmi M, Veronese N Assessing the quality of studies in meta-analyses: advantages and limitations of the Newcastle Ottawa Scale. World J Metaanal. 2017;5(4):80-84. doi:10.13105/wjma.v5.i4.80

[19] McCullagh PNJ Generalised Linear Models. Chapman and Hall; 1979.

[20] Kirkwood BR, Sterne JAC Essential Medical Statistics. 2nd ed Blackwell Publishing; 2003.

[21] Béjot Y, Bailly H, Graber M, Impact of the ageing population on the burden of stroke: the Dijon Stroke Registry. Neuroepidemiology. 2019;52(1-2):78-85. doi:10.1159/000492820 30602168

[22] Danese A, Stegagno C, Tomelleri G, ; Verostroke Group Clinical outcomes of secondary prevention strategies for young patients with cryptogenic stroke and patent foramen ovale. Acta Cardiol. 2017;72(4):410-418. doi:10.1080/00015385.2017.1307668 28705105

[23] Nezu T, Kitano T, Kubo S, Impact of D-dimer levels for short-term or long-term outcomes in cryptogenic stroke patients. J Neurol. 2018;265(3):628-636. doi:10.1007/s00415-018-8742-x 29372390

[24] Weimar C, Holle DN, Benemann J, ; German Stroke Study Collaboration Current management and risk of recurrent stroke in cerebrovascular patients with right-to-left cardiac shunt. Cerebrovasc Dis. 2009;28(4):349-356. doi:10.1159/000229553 19628936

[25] Diener HC, Sacco RL, Easton JD, ; RE-SPECT ESUS Steering Committee and Investigators Dabigatran for prevention of stroke after embolic stroke of undetermined source. N Engl J Med. 2019;380(20):1906-1917. doi:10.1056/NEJMoa1813959 31091372

[26] Meier B, Kalesan B, Mattle HP, ; PC Trial Investigators Percutaneous closure of patent foramen ovale in cryptogenic embolism. N Engl J Med. 2013;368(12):1083-1091. doi:10.1056/NEJMoa1211716 23514285

[27] Furlan AJ, Reisman M, Massaro J, ; CLOSURE I Investigators Closure or medical therapy for cryptogenic stroke with patent foramen ovale. N Engl J Med. 2012;366(11):991-999. doi:10.1056/NEJMoa1009639 22417252

[28] Kasner SE, Swaminathan B, Lavados P, ; NAVIGATE ESUS Investigators Rivaroxaban or aspirin for patent foramen ovale and embolic stroke of undetermined source: a prespecified subgroup analysis from the NAVIGATE ESUS trial. Lancet Neurol. 2018;17(12):1053-1060. doi:10.1016/S1474-4422(18)30319-3 30274772 PMC6662613

[29] Mas JL, Arquizan C, Lamy C, ; Patent Foramen Ovale and Atrial Septal Aneurysm Study Group Recurrent cerebrovascular events associated with patent foramen ovale, atrial septal aneurysm, or both. N Engl J Med. 2001;345(24):1740-1746. doi:10.1056/NEJMoa011503 11742048

[30] Serena J, Marti-Fàbregas J, Santamarina E, ; CODICIA, Right-to-Left Shunt in Cryptogenic Stroke Study; Stroke Project of the Cerebrovascular Diseases Study Group, Spanish Society of Neurology Recurrent stroke and massive right-to-left shunt: results from the prospective Spanish multicenter (CODICIA) study. Stroke. 2008;39(12):3131-3136. doi:10.1161/STROKEAHA.108.521427 18818401

[31] Moon J, Kang WC, Kim S, Comparison of outcomes after device closure and medication alone in patients with patent foramen ovale and cryptogenic stroke in Korean population. Yonsei Med J. 2016;57(3):621-625. doi:10.3349/ymj.2016.57.3.621 26996560 PMC4800350

[32] Paciaroni M, Agnelli G, Bertolini A, ; FORI (Foramen Ovale Registro Italiano) Investigators Risk of recurrent cerebrovascular events in patients with cryptogenic stroke or transient ischemic attack and patent foramen ovale: the FORI (Foramen Ovale Registro Italiano) study. Cerebrovasc Dis. 2011;31(2):109-116. doi:10.1159/000321334 21088390

[33] Horner S, Niederkorn K, Gattringer T, Management of right-to-left shunt in cryptogenic cerebrovascular disease: results from the observational Austrian Paradoxical Cerebral Embolism Trial (TACET) registry. J Neurol. 2013;260(1):260-267. doi:10.1007/s00415-012-6629-9 22865239

[34] Harrer JU, Wessels T, Franke A, Lucas S, Berlit P, Klötzsch C Stroke recurrence and its prevention in patients with patent foramen ovale. Can J Neurol Sci. 2006;33(1):39-47. doi:10.1017/S0317167100004674 16583720

[35] Lee JY, Song JK, Song JM, Association between anatomic features of atrial septal abnormalities obtained by omni-plane transesophageal echocardiography and stroke recurrence in cryptogenic stroke patients with patent foramen ovale. Am J Cardiol. 2010;106(1):129-134. doi:10.1016/j.amjcard.2010.02.025 20609660

[36] Wahl A, Jüni P, Mono ML, Long-term propensity score-matched comparison of percutaneous closure of patent foramen ovale with medical treatment after paradoxical embolism. Circulation. 2012;125(6):803-812. doi:10.1161/CIRCULATIONAHA.111.030494 22238228

[37] Schuchlenz HW, Weihs W, Berghold A, Lechner A, Schmidt R Secondary prevention after cryptogenic cerebrovascular events in patients with patent foramen ovale. Int J Cardiol. 2005;101(1):77-82. doi:10.1016/j.ijcard.2004.03.005 15860387

[38] Casaubon L, McLaughlin P, Webb G, Yeo E, Merker D, Jaigobin C Recurrent stroke/TIA in cryptogenic stroke patients with patent foramen ovale. Can J Neurol Sci. 2007;34(1):74-80. doi:10.1017/S0317167100005825 17352351

[39] Anzola GP, Zavarize P, Morandi E, Rozzini L, Parrinello G Transcranial Doppler and risk of recurrence in patients with stroke and patent foramen ovale. Eur J Neurol. 2003;10(2):129-135. doi:10.1046/j.1468-1331.2003.00561.x 12603287

[40] Cerrato P, Priano L, Imperiale D, Recurrent cerebrovascular ischaemic events in patients with interatrial septal abnormalities: a follow-up study. Neurol Sci. 2006;26(6):411-418. doi:10.1007/s10072-006-0524-z 16601933

[41] Cujec B, Mainra R, Johnson DH Prevention of recurrent cerebral ischemic events in patients with patent foramen ovale and cryptogenic strokes or transient ischemic attacks. Can J Cardiol. 1999;15(1):57-64. 10024860

[42] Hanna JP, Sun JP, Furlan AJ, Stewart WJ, Sila CA, Tan M Patent foramen ovale and brain infarct: echocardiographic predictors, recurrence, and prevention. Stroke. 1994;25(4):782-786. doi:10.1161/01.STR.25.4.782 8160221

[43] Hausmann D, Mügge A, Daniel WG Identification of patent foramen ovale permitting paradoxic embolism. J Am Coll Cardiol. 1995;26(4):1030-1038. doi:10.1016/0735-1097(95)00288-9 7560596

[44] Thanopoulos BV, Dardas PD, Karanasios E, Mezilis N Transcatheter closure versus medical therapy of patent foramen ovale and cryptogenic stroke. Catheter Cardiovasc Interv. 2006;68(5):741-746. doi:10.1002/ccd.20868 17039525

[45] Shariat A, Yaghoubi E, Farazdaghi M, Aghasadeghi K, Borhani Haghighi A Comparison of medical treatments in cryptogenic stroke patients with patent foramen ovale: a randomized clinical trial. J Res Med Sci. 2013;18(2):94-98. 23914208 PMC3724385

[46] Bogousslavsky J, Garazi S, Jeanrenaud X, Aebischer N, Van Melle G; Lausanne Stroke with Paradoxal Embolism Study Group Stroke recurrence in patients with patent foramen ovale: the Lausanne Study. Neurology. 1996;46(5):1301-1305. doi:10.1212/WNL.46.5.1301 8628471

[47] Comess KA, DeRook FA, Beach KW, Lytle NJ, Golby AJ, Albers GW Transesophageal echocardiography and carotid ultrasound in patients with cerebral ischemia: prevalence of findings and recurrent stroke risk. J Am Coll Cardiol. 1994;23(7):1598-1603. doi:10.1016/0735-1097(94)90662-9 8195520

[48] De Castro S, Cartoni D, Fiorelli M, Morphological and functional characteristics of patent foramen ovale and their embolic implications. Stroke. 2000;31(10):2407-2413. doi:10.1161/01.STR.31.10.2407 11022072

[49] Eriksson SE Secondary prophylactic treatment and long-term prognosis after TIA and different subtypes of stroke: a 25-year follow-up hospital-based observational study. Brain Behav. 2016;7(1):e00603. doi:10.1002/brb3.603 28127521 PMC5256186

[50] Faggiano P, Frattini S, Piovesana P, Low cerebrovascular event rate in subjects with patent foramen ovale and different clinical presentations: results from a prospective non randomized study on a population including patients with and without patent foramen ovale closure. Int J Cardiol. 2012;156(1):47-52. doi:10.1016/j.ijcard.2010.10.032 21112103

[51] Morais LA, Sousa L, Fiarresga A, RoPE score as a predictor of recurrent ischemic events after percutaneous patent foramen ovale closure. Int Heart J. 2018;59(6):1327-1332. doi:10.1536/ihj.17-489 30305578

[52] Putaala J, Nieminen T, Haapaniemi E, Undetermined stroke with an embolic pattern—a common phenotype with high early recurrence risk. Ann Med. 2015;47(5):406-413. doi:10.3109/07853890.2015.1057612 26224200

[53] Stone DA, Godard J, Corretti MC, Patent foramen ovale: association between the degree of shunt by contrast transesophageal echocardiography and the risk of future ischemic neurologic events. Am Heart J. 1996;131(1):158-161. doi:10.1016/S0002-8703(96)90065-4 8554004

[54] Mirzada N, Ladenvall P, Hansson PO, Eriksson P, Dellborg M Recurrent stroke in patients with patent foramen ovale: an observational prospective study of percutaneous closure of PFO versus non-closure. Int J Cardiol. 2015;195:293-299. doi:10.1016/j.ijcard.2015.05.088 26056962

[55] Elmariah S, Furlan AJ, Reisman M, ; CLOSURE I Investigators Predictors of recurrent events in patients with cryptogenic stroke and patent foramen ovale within the CLOSURE I (Evaluation of the STARFlex Septal Closure System in Patients With a Stroke and/or Transient Ischemic Attack Due to Presumed Paradoxical Embolism Through a Patent Foramen Ovale) trial. JACC Cardiovasc Interv. 2014;7(8):913-920. doi:10.1016/j.jcin.2014.01.170 25147037

[56] Furlan AJ; CLOSURE I Investigators PFO closure: CLOSURE. Stroke. 2013;44(6)(suppl 1):S45-S47. doi:10.1161/STROKEAHA.113.000975 23709727

[57] Pezzini A, Grassi M, Lodigiani C, ; Italian Project on Stroke in Young Adults (IPSYS) Investigators Propensity score–based analysis of percutaneous closure versus medical therapy in patients with cryptogenic stroke and patent foramen ovale: the IPSYS Registry (Italian Project on Stroke in Young Adults). Circ Cardiovasc Interv. 2016;9(9):e003470. doi:10.1161/CIRCINTERVENTIONS.115.003470 27582111

[58] Homma S, Sacco RL, Di Tullio MR, Sciacca RR, Mohr JP; PFO in Cryptogenic Stroke Study (PICSS) Investigators Effect of medical treatment in stroke patients with patent foramen ovale: patent foramen ovale in Cryptogenic Stroke Study. Circulation. 2002;105(22):2625-2631. doi:10.1161/01.CIR.0000017498.88393.44 12045168

[59] Anderson FA Jr, Wheeler HB, Goldberg RJ, A population-based perspective of the hospital incidence and case-fatality rates of deep vein thrombosis and pulmonary embolism. The Worcester DVT Study. Arch Intern Med. 1991;151(5):933-938. doi:10.1001/archinte.1991.00400050081016 2025141

[60] Hagen PT, Scholz DG, Edwards WD Incidence and size of patent foramen ovale during the first 10 decades of life: an autopsy study of 965 normal hearts. Mayo Clin Proc. 1984;59(1):17-20. doi:10.1016/S0025-6196(12)60336-X 6694427

[61] Merkler AE, Gialdini G, Yaghi S, Safety outcomes after percutaneous transcatheter closure of patent foramen ovale. Stroke. 2017;48(11):3073-3077. doi:10.1161/STROKEAHA.117.018501 28939677 PMC5699514

[62] Emberson J, Lees KR, Lyden P, ; Stroke Thrombolysis Trialists’ Collaborative Group Effect of treatment delay, age, and stroke severity on the effects of intravenous thrombolysis with alteplase for acute ischaemic stroke: a meta-analysis of individual patient data from randomised trials. Lancet. 2014;384(9958):1929-1935. doi:10.1016/S0140-6736(14)60584-5 25106063 PMC4441266

[63] Rothwell PM, Eliasziw M, Gutnikov SA, Warlow CP, Barnett HJM; Carotid Endarterectomy Trialists Collaboration Endarterectomy for symptomatic carotid stenosis in relation to clinical subgroups and timing of surgery. Lancet. 2004;363(9413):915-924. doi:10.1016/S0140-6736(04)15785-1 15043958

[64] Wiktor DM, Carroll JD The case for selective patent foramen ovale closure after cryptogenic stroke. Circ Cardiovasc Interv. 2018;11(3):e004152. doi:10.1161/CIRCINTERVENTIONS.117.004152 29870380

[65] Mojadidi MK, Roberts SC, Winoker JS, Accuracy of transcranial Doppler for the diagnosis of intracardiac right-to-left shunt: a bivariate meta-analysis of prospective studies. JACC Cardiovasc Imaging. 2014;7(3):236-250. doi:10.1016/j.jcmg.2013.12.011 24560213

