# Supplementary material for: Prognosis of Cryptogenic Stroke With Patent Foramen Ovale at Older Ages and Implications for Trials: A Population-Based Study and Systematic Review
Source: JAMA Neurol. 2020 Jul 6;77(10):1–9. doi: 10.1001/jamaneurol.2020.1948 (PMC7550974; doi:10.1001/jamaneurol.2020.1948)
Supplement: Supplement. — eMethods. OxVasc Methodology eResults. Characteristics of Studies Included in the Systematic Review eFigure 1. PRISMA Flow Chart eTable 1. Data Extraction Forms With Included Papers eTable 2. Quality Assessment for Included Studies With the Newcastle-Ottawa Scale eFigure 2. Subgroup Analysis on the Absolute Risk of Recurrent Ischaemic Stroke Calculated on the 23 Studies Reporting This Outcome eTable 3. Sample Size Calculation for Trials of PFO Closure Versus Medical Treatment Only After Cryptogenic TIA/Stroke in Patients ≥60 Years of Age [file jamaneurol-e201948-s001.pdf]

Supplementary Online Content

Mazzucco S, Li L, Rothwell PM. Prognosis of cryptogenic stroke with patent foramen ovale at older ages and implications for trials: a population-based study and systematic review. *JAMA Neurol*. Published online June 29, 2020. doi:10.1001/jamaneurol.2020.1948

eMethods. OXVASC Methodology; Search strategy

eResults. Systematic Review Bibliography; Characteristics of Studies Included in the Systematic Review

eFigure 1. PRISMA Flow Chart

eTable 1. Data Extraction Forms With Included Papers

eTable 2. Quality Assessment for Included Studies With the Newcastle Ottawa Scale

eFigure 2. Subgroup Analysis on the Absolute Risk of Recurrent Ischaemic Stroke Calculated on the 23 Studies Reporting This Outcome

eTable 3. Sample Size Calculation for Trials of PFO Closure Versus Medical Treatment Only After Cryptogenic TIA/Stroke in Patients ≥60 Years of Age

This supplementary material has been provided by the authors to give readers additional information about their work.

© 2020 American Medical Association. All rights reserved.

eMethods: OXVASC methodology

Study population

The Oxford Vascular Study (OXVASC) is a prospective, population-based cohort study of all incident acute vascular events in all territories (transient ischaemic attack, stroke, acute coronary and peripheral vascular events).[1,2]

During the period of the current substudy, the OXVASC study population consisted of all 92,728 individuals, irrespective of age, registered with 100 general practitioners (GPs) in nine general practices in Oxfordshire, UK. In the UK, general practices provide primary health care for registered individuals and hold a lifelong record of all medical consultations (from the National Health Service [NHS] and private health care), and details of treatments, blood pressure, and investigations. In Oxfordshire, an estimated 97% of the true residential population is registered with a general practice, with most non-registered individuals being young students. All participating practices held accurate age-sex patient registers, and allowed regular searches of their computerised diagnostic coding systems. The practices had all collaborated on a previous population-based study, for which they were originally selected to be representative of the urban and rural mix and the deprivation range of Oxfordshire as a whole.[3] Based on the index of multiple deprivation (IMD), the population was less deprived than the rest of England, but had a broad range of deprivation.

The OXVASC population is 94% white people, 3% Asian, 2% Chinese, and 1% Afro-Caribbean.[4] The proportion of whites is similar to that of the UK as a whole (88% white) and to many other western countries (Australia - 90%; France - 91%; Germany - 93.9%).

Case ascertainment

After a 3-month pilot study, the study started on April 1, 2002, and is ongoing. Ascertainment combined prospective daily searches for acute events (hot pursuit) and retrospective searches of hospital-care and primary-care administrative and diagnostic coding data (cold pursuit).

Hot pursuit was based on:

1. A daily (weekdays only), urgent open-access "TIA clinic" to which participating general practitioners (GPs) and the local accident and emergency department (A&E) send all individuals with suspected TIA or stroke whom they would not normally admit to hospital, with alternative on-call review provision at weekends. Patients too frail to attend are assessed at their residence by a study nurse or doctor.
2. Daily searches and case note review of admissions to the Emergency Assessment Unit, Medical Short Stay Unit, Coronary Care Unit and Cardiothoracic Critical Care Unit, Cardiology, Cardiothoracic, and Vascular Surgery wards, Acute Stroke Unit, Neurology ward and all other general wards when indicated.
3. Daily searches of the local A&E and eye hospital attendance registers.
4. Daily identification via the Bereavement Office of patients dead on arrival at hospital or who died soon after.
5. Daily searches of lists of all patients from the study population in whom a troponin-I level had been requested.
6. Daily assessment of all patients undergoing diagnostic coronary, carotid and peripheral angiography, angioplasty, stenting or vascular surgical procedures in any territory to identify both total burden of vascular invention and any potential missed prior acute events.

Cold pursuit procedures were:

1. Frequent visits to the study practices and monthly searches of practice diagnostic codes.
2. Monthly practice-specific list of all patients admitted to all acute and community NHS hospitals.
3. Monthly listings of all referrals for brain or carotid imaging studies performed in local hospitals.
4. Monthly reviews of all death certificates and coroners reports to review out-of-hospital deaths.
5. Practice-specific listings of all ICD-10 death codes from the local Department of Public Health.

Patients found on GP practice searches who have an event whilst temporarily out of Oxfordshire are included, but visitors who were not registered with one of the study practices are excluded. A study clinician assessed

© 2020 American Medical Association. All rights reserved.

patients as soon as possible after the event in the hospital or at home. Informed consent was sought, if possible, or assent was obtained from a relative.

Data are collected using event-specific forms, for TIA and stroke, acute coronary syndrome or acute peripheral vascular events. Standardised clinical history and cardiovascular examination are recorded. Information recorded from the patient, their hospital records and their general practice records includes details of the clinical event, medication, past medical history, all investigations relevant to their admission (including blood results, electrocardiography, brain imaging and vascular imaging-duplex ultrasonography, CT-angiography, MR-angiography or DSA) and all interventions occurring subsequent to the event.

If a patient died before assessment, we obtained an eyewitness account of the clinical event and reviewed any relevant records. If death occurred outside the hospital or before investigation, the autopsy result was reviewed. Clinical details are sought from primary care physicians or other clinicians on all deaths of possible vascular aetiology.

All surviving TIA and stroke patients are followed-up face-to-face at 1, 6, 12, 60 and 120 months after the initial event by a research nurse or physician and all recurrent vascular events were recorded together with the relevant clinical details and investigations. If face-to-face follow up is not possible, telephone follow-up is performed or enabled via the general practitioner. All recurrent vascular events that presented to medical attention would also be identified acutely by ongoing daily case ascertainment within OXVASC. If a recurrent vascular event was suspected at a follow-up visit or referred by the GPs to clinic or admitted, the patient was re-assessed and investigated by a study physician.

Definitions of events

Although new definitions for stroke and TIA have been suggested recently,[4,5] in order to enable comparison with previous studies, the classic definitions of TIA and stroke are used throughout.[6] A stroke is defined as rapidly developing clinical symptoms and/or signs of focal, and at time global (applied to patients in deep coma and to those with subarachnoid haemorrhage), loss of brain function, with symptoms lasting more than 24 hours or leading to death, with no apparent cause other than that of vascular origin.[6] A TIA is an acute loss of focal brain or monocular function with symptoms lasting less than 24 hours and which is thought to be caused by inadequate cerebral or ocular blood supply as a result of arterial thrombosis, low flow or embolism associated with arterial, cardiac or haematological disease.[4] All diagnoses were reviewed by a senior neurologist (PMR). With the high rate (97%) of imaging or autopsy in OXVASC, strokes of unknown type were coded as ischaemic.

References

1. Rothwell PM, Coull AJ, Giles MF, et al. Change in stroke incidence, mortality, case-fatality, severity, and risk factors in Oxfordshire, UK from 1981 to 2004 (Oxford Vascular Study). Lancet 2004; 363: 1925–33.
2. Rothwell PM, Coull AJ, Silver LE, Fairhead JF, Giles MF, Lovelock CE, Redgrave JNE, Bull LM, Welch SJV, Cuthbertson FC, Binney LE, Gutnikov SA, Anslow P, Banning AP, Mant D, Mehta Z for the Oxford Vascular Study. Population-based study of event-rate, incidence, case fatality and mortality for all acute vascular events in all arterial territories (Oxford Vascular Study). Lancet 2005; 366: 1773-83.
3. Bamford J, Sandercock P, Dennis M, Burn J, Warlow C. A prospective study of acute cerebrovascular disease in the community: the Oxfordshire Community Stroke Project--1981-86. 2. Incidence, case fatality rates and overall outcome at one year of cerebral infarction, primary intracerebral and subarachnoid haemorrhage. *J Neurol Neurosurg Psychiatry* 1990;53:16-22.
4. Easton JD, Saver JL, Albers GW, et al. Definition and evaluation of transient ischemic attack: a scientific statement for healthcare professionals from the American Heart Association/American Stroke Association Stroke Council; Council on Cardiovascular Surgery and Anesthesia; Council on Cardiovascular Radiology and Intervention; Council on Cardiovascular Nursing; and the Interdisciplinary Council on Peripheral Vascular Disease. The American Academy of Neurology affirms the value of this statement as an educational tool for neurologists. *Stroke* 2009;40:2276-2293.
5. Sacco RL, Kasner SE, Broderick JP, et al. An updated definition of stroke for the 21st century: a statement for healthcare professionals from the American Heart Association/American Stroke Association. *Stroke* 2013;44:2064-2089.
6. Hatano S. Experience from a multicentre stroke register: a preliminary report. *Bulletin of the World Health Organization* 1976;54:541-553.

© 2020 American Medical Association. All rights reserved.

Search strategy

We carried out a systematic review according to the PRISMA criteria and registered the protocol on PROSPERO website (number CRD42019127020).
We searched MEDLINE and Web of Science for articles published before March 31st 2019, using the terms *“stroke”, “cryptogenic stroke”, “stroke of undetermined aetiology”, “embolic stroke of undetermined source”, “foramen ovale”, “PFO”, “atrial septal abnormality”, “interatrial septal abnormality”, “right-to-left shunt”, “prognosis”, “recurrent stroke”,* as follows:

[((“stroke”[Mesh]) OR “cryptogenic stroke” OR “stroke of undetermined aetiology” OR “embolic stroke of undetermined source”) AND (("Foramen Ovale, Patent"[Mesh]) OR PFO OR “atrial septal abnormality” OR “interatrial septal abnormality” OR “right-to-left shunt”)] AND [("Prognosis"[Mesh]) OR “recurrent stroke”]

© 2020 American Medical Association. All rights reserved.

eResults: Systematic Review Bibliography

S1. Carroll JD, Saver JL, Thaler DE, et al. Closure of Patent Foramen Ovale versus Medical Therapy after Cryptogenic Stroke. *N Engl J Med* 2013;368:1092-100.

S2. Saver JL, Carroll JD, Thaler DE, et al. Long-Term Outcomes of Patent Foramen Ovale Closure or Medical Therapy after Stroke. *N Engl J Med* 2017;377:1022-32.

S3. Mas JL, Derumeaux G, Guillon B, et al. Patent Foramen Ovale Closure or Anticoagulation vs. Antiplatelets after Stroke. *N Engl J Med 2017*;377:1011-21.

S4. Søndegaard L, Kasner SE, Rhodes JF, et al. Patent Foramen Ovale Closureor Antiplatelet Therapy for Cryptogenic Stroke. *N Engl J Med* 2017; 377:1033-42.

S5. Lee PH, Song JK, Kim JS, et al. Cryptogenic Stroke and High-Risk Patent Foramen Ovale The DEFENSE-PFO Trial. *JACC* 2018;71:335-42.

S6. Homma S, Sacco RL, Di Tullio MR, Sciacca RR, Mohr JP, MD; for PICSS Investigators. Age As a Determinant of Adverse Events in Medically Treated Cryptogenic Stroke Patients With Patent Foramen Ovale. *Stroke* 2004;35:2145-49.

S7. Mas JL, Zuber M, for the French Study Group on Patent Foramen Ovale and Atrial Septal Aneurysm. Recurrent cerebrovascular events in patients with patent foramen ovale, atrial septal aneurysm or both and cryptogenic stroke or transient ischemic attack. *Am H J* 1995;130:1083-8.

S8. Anzola GP, Zavarise P, Morandi E, Rozzini L, Parrinello G. Transcranial Doppler and risk of recurrence in patients with stroke and patent foramen ovale. *Eur J Neurol* 2003; 10:129-135.

S9. Cerrato P, Priano L, Imperiale D, et al. Recurrent cerebrovascular ischaemic events in patients with interatrial septal abnormalities: a follow-up study. *Neurol Sci* 2006;26:411-18.

S10. Cujec B, Mainra R, Johnson DH. Prevention of recurrent cerebral ischemic events in patients with patent foramen ovale and cryptogenic strokes or transient ischaemic attacks. *Can J Cardiol* 1999;15:57-64.

S11. Hanna JP, Sun JP, Furlan AJ, Stewart WJ, Sila CA, Tan M. Patent Foramen Ovale and brain infarct. Echocardiographic predictors, Recurrence and prevention. *Stroke* 1994; 25:782-786.

S12. Hausmann D, Mügge A, Daniel WG. Identification of Patent Foramen Ovale permitting paradoxical embolism. *J Am Coll Cardiol* 1995; 26:1030-8.

S13. Thanopoulos BV, Dardas PD, Karanasios E, Mezilis N. Transcatheter closue versus medical therapy of Patent Foramen Ovale and Cryptogenic stroke. *Catheter Cardio Inte* 2006; 68:741-46.

S14. Shariat A, Yaghoubi E, Farazdaghi M, Aghasadeghi K, Borhani Haghighi A. Comparison of medical treatments in cryptogenic stroke patients with patent foramen ovale: A randomized clinical trial. *J Res Med Sci* 2013;18: 94–98.

S15. Bogousslavsky J, Garazi S, Jeanrenaud X, Aebischer N, Van Melle G, for the Lausanne Stroke with Paradoxal Embolism Study Group. Stroke recurrence in patients with patent foramen ovale: The Lausanne Study. *Neurology* 1996;46:1301-305.

S16. Comess K, Derook FA, Beach KW, Lytle NJ, Golby AJ, Albers GW. Transesophageal Echocardiography and Carotid Ultrasound in Patients With Cerebral Ischemia: Prevalence of Findings and Recurrent Stroke Risk. *J Am Coll Cardiol* 1994;23:1598-603.

S17. De Castro S, Cartoni D, Fiorelli M, et al. Morphological and Functional Characteristics of Patent Foramen Ovale and Their Embolic Implications. *Stroke* 2000;**31**:2407-13.

© 2020 American Medical Association. All rights reserved.

S18. Eriksson SE. Secondary prophylactic treatment and long- term prognosis after TIA and different subtypes of stroke. A 25- year follow- up hospital- based observational study. Brain and Behavior 2017; 7: e00603.

S19. Faggiano P, Frattini S, Piovesana G, et al. Low cerebrovascular event rate in subjects with Patent Foramen Ovale and different clinical presentations. Results from a prospective non randomised study on a population including patients with and without Patent Foramen Ovale closure. *Int J Cardiol* 2012;156:47-52.

S20. Morais LA, de Sousa L, Fiarresga A, et al. RoPE Score as a Predictor of Recurrent Ischemic Events After Percutaneous Patent Foramen Ovale Closure. Int Heart J 2018; 59:1327-32.

S21. Putaala J, Nieminen T, Haapaniemi E, et al. Undetermined stroke with an embolic pattern—a common phenotype with high early recurrence risk. Annals of Medicine, 2015;47:406-13.

S22. Stone DA, Godard J, Corretti MC, et al. Patent foramen ovale: Association between the degree of shunt by contrast transesophageal echocardiography and the risk of future ischemic neurologic events. *Am Heart J* 1996;131:158-61.

S23. Mirzada N, Ladenvall P, Hansson PO, Eriksson P, Dellborg M. Recurrent stroke in patients with Patent Foramen Ovale: an observational prospective study of percutaneous closure of PFO versus non closure. *Int J Cardiol* 2015;195:293-99.

S24. Elmariah S, Furlan AJ, Reisman M, et al. Predictors of Recurrent Events in Patients With Cryptogenic Stroke and Patent Foramen Ovale Within the CLOSURE I (Evaluation of the STARFlex Septal Closure System in Patients With a Stroke and/or Transient Ischemic Attack Due to Presumed Paradoxical Embolism Through a Patent Foramen Ovale) Trial. JACC: Cardiovascular Interventions 2014;7:913-20.

S25. Furlan, AJ; for the CLOSURE I Investigators. PFO Closure: CLOSURE. Stroke. 2013;44:S45-S47.

S26. Pezzini A, Grassi M, Lodigiani C, Patella R, Gandolfo C, Zini A et al. Propensity Score-Based Analysis of Percutaneous Closure Versus Medical Therapy in Patients With Cryptogenic Stroke and Patent Foramen Ovale: The IPSYS Registry (Italian Project on Stroke in Young Adults). Circ Cardiovasc Interv. 2016;9:e003470.

S27. Danese A, Stegagno C, Tomelleri GP, et al. Clinical outcomes of secondary prevention strategies for young patients with cryptogenic stroke and patent foramen ovale. *Acta Cardiologica* 2017;72:410-418.

S28. Homma S, Sacco RL, Di Tullio MR, et al. Effect of Medical Treatment in Stroke Patients With Patent Foramen Ovale Patent Foramen Ovale in Cryptogenic Stroke Study. *Circulation* 2002;**105**:2625-2631.

S29. Nezu T, Kitano T, Kubo S, et al. D-dimer levels for short-term or long-term outcomes in cryptogenic stroke patients. J Neurol 2018;265:628-36.

S30. Weimar C, Holle DN, Benemann J, et al. Current Management and Risk of Recurrent Stroke in Cerebrovascular Patients with Right-to-Left Cardiac Shunt. *Cerebrovasc Dis* 2009;28:349–56.

S31. Kasner SE, Swaminathan B, Lavados P, et al. Rivaroxaban or aspirin for patent foramen ovale and embolic stroke of undetermined source: a prespecified subgroup analysis from the NAVIGATE ESUS trial. *Lancet Neurol* 2018;17:1053-60.

S32. Mas JL, Arquizan C, Lamy C, et al. Recurrent cerebrovascular events associated with patent foramen ovale, atrial septal aneurysm or both. N Engl J Med 2001;345:1740-6.

S33. Serena J, Marti-Fa`bregas J, Santamarina E, et al. Recurrent Stroke and Massive Right-to-Left Shunt Results From the Prospective Spanish Multicenter (CODICIA) Study. *Stroke*. 2008;39:3131-36.

S34. Harrer JU, Wessels T, Franke A, Lucas S, Berlit P, Klötzsch C. Stroke Recurrence and its Prevention in Patients with Patent Foramen Ovale. *Can J Neurol Sci* 2006;33:39-47.

© 2020 American Medical Association. All rights reserved.

S35. Meier B, Kalesan B, Mattle HP, et al. Percutaneous Closure of Patent Foramen Ovale in Cryptogenic Embolism. *N Engl J Med* 2013;368:1083-91.

S36. Moon J, WC Kang, Kim S, et al. Comparison of Outcomes after device Closure and Medication Alone in Patients with Patent Foramen Ovale and Cryptogenic Stroke in Korean Population. *Yonsei Med* J 2016;57:621-25.

S37. Lee JY, Song JK, Song JM, et al. Association Between Anatomic Features of Atrial Septal Abnormalities Obtained by Omni-Plane Transesophageal Echocardiography and Stroke Recurrence in Cryptogenic Stroke Patients with Patent Foramen Ovale. *Am J Cardiol* 2010;106:129 –34.

S38. Paciaroni M, Agnelli G, Bertolini A, et al. Risk of Recurrent Cerebrovascular Events in Patients with Cryptogenic Stroke or Transient Ischemic Attack and Patent Foramen Ovale: The FORI (Foramen Ovale Registro Italiano) Study. *Cerebrovasc Dis* 2011;31:109–16.

S39. Furlan AJ, Reisman M, Massaro J, et al. Closure or Medical Therapy for Cryptogenic Stroke with Patent Foramen Ovale. N Engl J Med 2012;366:991-9.

S40. Wahl A, Jüni P, Mono ML, et al. Long-Term Propensity Score–Matched Comparison of Percutaneous Closure of Patent Foramen Ovale With Medical Treatment After Paradoxical Embolism. *Circulation*. 2012;125:803-12

S41. Casaubon L, McLaughlin P, Webb G, Yeo E, Merker D, Jaigobin C. Recurrent Stroke/TIA in Cryptogenic Stroke Patients with Patent Foramen Ovale. Can J Neurol Sci 2007; 34:74-80.

S42. Horner S, Niederkorn K, Gattringer T, et al. Management of right-to-left shunt in cryptogenic cerebrovascular disease: results from the observational Austrian paradoxical cerebral embolism trial (TACET) registry. *J Neurol* 2013;260:260–67.

S43. Schuchlenz HW, Weihs W, Berghold A, Lechner A, Schmidt R. Secondary prevention after cryptogenic cerebrovascular events in patients with patent foramen ovale. International Journal of Cardiology 2005;101:77– 82.

S44. Diener HC, Sacco RL, Easton JD, et al. Dabigatran for Prevention of Stroke after Embolic Stroke of Undetermined Source. *N Engl J Med* 2019;380:1906-17.

S45. Mazzucco S, Bovi P, Carletti M, et al. A model of multi-disciplinary approach to the diagnosis and treatment of young patients with cryptogenic stroke and patent foramen ovale. *Cardiol Young* 2012;22,327–334.

S46. Nedeltchev K, Arnold M, Wahl A, et al. Outcome of patients with cryptogenic stroke and patent foramen ovale. J Neurol Neurosurg Psychiatry *2002*;72:347–350.

S47. Windecker S, Wahl A, Nedeltchev K, et al. Comparison of Medical Treatment With Percutaneous Closure of Patent Foramen Ovale in Patients With Cryptogenic Stroke. *JACC* 2004;44:750–8.

© 2020 American Medical Association. All rights reserved.

eResults: Characteristics of studies included in the Systematic Review

Of 577 potentially eligible records found by the systematic review, we identified 28 eligible papers reporting on 23 studies (nine trials and 14 observational studies) including 4889 patients with cryptogenic stroke/TIA and PFO on medical treatment alone (PRISMA Flow Chart, Figure I in the Supplement). Non-eligible studies included: observational studies[S8-S13] and one randomised trial (47 patients with cryptogenic stroke/TIA randomised to either warfarin or aspirin)[S14] with a population below the threshold for inclusion (<100 cryptogenic stroke/TIA patients enrolled); observational studies on an unsuitable population (mostly retrospective studies selecting patients on the basis of echocardiographic finding of a PFO, where the analysis included non-stroke patients as well, or stroke/TIA patients with both cryptogenic and non-cryptogenic stroke/TIA);[S15-S23] secondary publications of an included study;[S24,S25] and one paper that reported only composite outcomes of stroke/TIA and peripheral embolism.[S26] We contacted eight authors and obtained unpublished data from four.[S6,S27-S30]

In the studies reporting data for the population with PFO (22/23 studies), 1738/4224 (41.15%) of patients were females; mean (SD) age was 50.41 (8.40) years; mean (SD) age for closure trials was significantly lower - 46.51 (3.76) years vs 51.79 (9.22) in other cohorts, (p=0.0006). Only 6 studies[S3,S6,S29, S31-S33] reported on baseline disability, with 1299/1604 (80.1%) of enrolled patients having mRS ≤2. In the 14 studies reporting on RLS size,[S2-S4,S27,S30,S31,S33-S40] 1602/3040 (52.70%) of patients had a small/medium RLS, but this varied widely, ranging from only 5% of patients with mild-to-moderate RLS and associated ASA in the antiplatelet group of the CLOSE trial[S3] to 86.2% of patients with mild-to-moderate RLS in the observational study by Lee et al.[S37] In the 18 studies reporting on the presence of ASA, 744/2901 (25.65%) patients overall had associated ASA.[S2,S3,S5,S7,S25,S27,S30-S35,S37,S38,S40-S43]

Across studies reporting on the type of anti-thrombotic treatment, 1470/4618 patients (31.83%) were on anticoagulation. Anticoagulants included vitamin K antagonists, heparin and, in the more recent trials, direct anticoagulants.[S3,S31,S44] Antiplatelet regimens included aspirin, thienopyridines, or a combination of Aspirin and extended-release dipyridamole, clopidogrel, or cilostazol[S5] (eTable 1).

© 2020 American Medical Association. All rights reserved.

eFigure 1: PRISMA flow chart

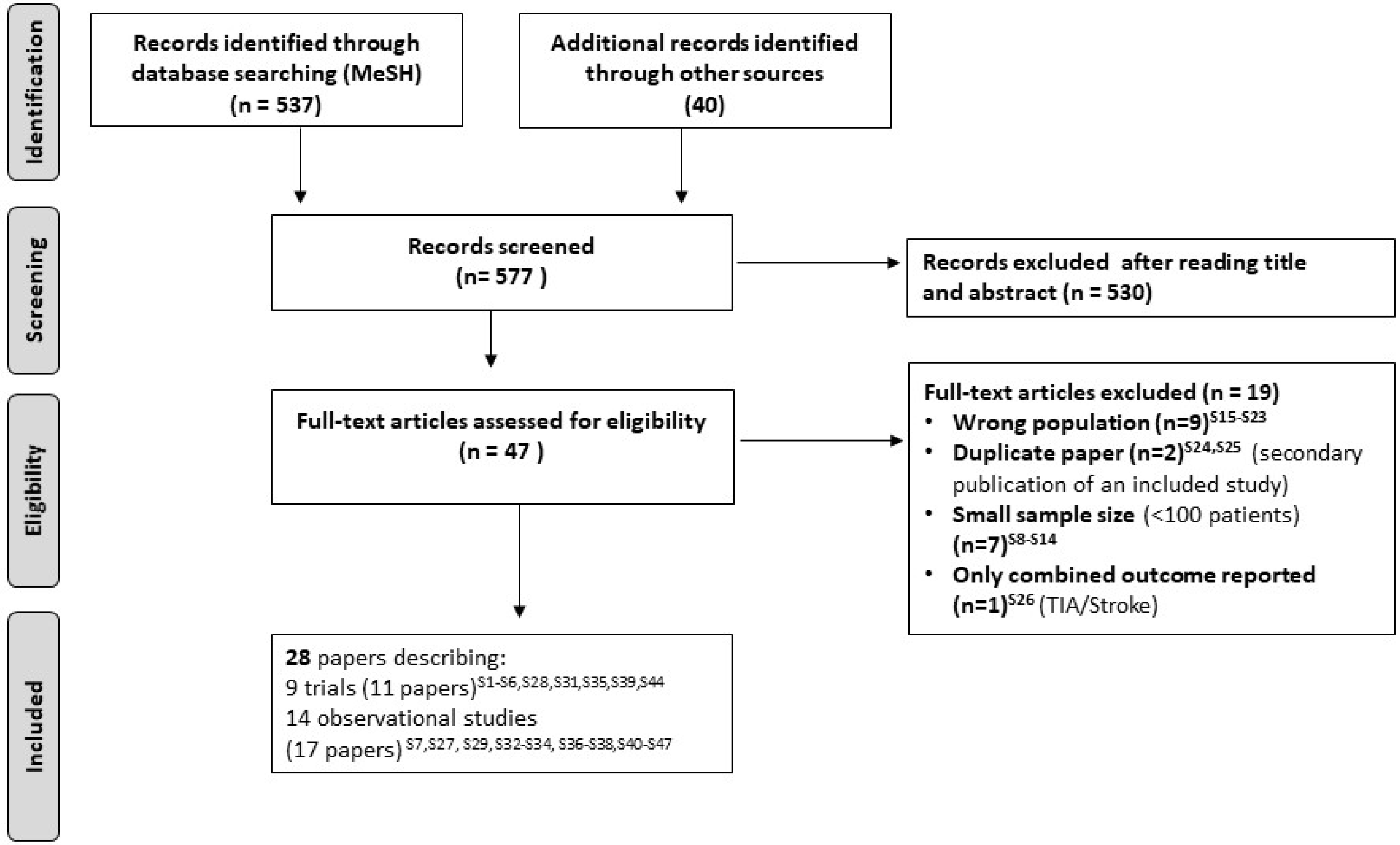

© 2020 American Medical Association. All rights reserved.

eTable 1:

Data extraction forms with included papers. mRS: modified Rankin Scale; TTE: trans-thoracic echocardiogram; TOE: Trans-Oesophageal Echocardiogram,; TCD: Transcranial Doppler; NR: Not reported; FU: Follow-up; [&]Assessment of index event was considered complete if the diagnostic work-up included at least brain imaging, ECG, and extra-cranial imaging; ^Direct FU assessment: face-to-face medical assessment triggered by a suspected recurrence; *Duplicate study

© 2020 American Medical Association. All rights reserved.

Paper	Recruitment period	Study type	Setting of enrolment	Cryptogenic/ Determined aetiology	Cryptogenic PFO+/ PFO-	Age cut-off; mean age	Mean (SD) FU duration (months)	Complete[&]/ incomplete/NR assessment for index event	Method of PFO diagnosis	TOAST	>24 h ECG monitoring for index event?	Type of index event	Baseline mRS	Medical treatment of cryptogenic PFO+ after index event	% Major bleeds reported	Type of recurrent event	Direct[^]/ indirect/ NR assessment for recurrent event
**Mas JL, 1995**	1898-1991	Retrospective observational multicentre French cohort of cryptogenic IS/TIA with PFO, ASA or both, who underwent TOE	Neurology centres	132/50	107/25 (with ASA only)	≤45-60 (variable by centres); mean age 36.9±10.4, 43.8±9.1	22.6±16	Complete	TOE	Unspecified	No (24h recording in 70% of pts)	Stroke (111) and TIA (21)	NR	Of the 132 pts, 59 (44.7%) APTs/ 33 (25%) ACO (ACO in 14.5% of isolated PFO vs 44% isolated ASA, vs 31.6% both) APTs were Aspirin 250-500 mg, 3 pts on Ticlopidine 500 mg; 6 patients received no treatment, 34 received AP/AC during part of the FU.	2 non fatal bleed in ACO	Ischaemic stroke (2),<br>TIA (4)	Indirect , plus telephone call
**Mas JL, 2001**	1996-1998	Observational , multicentre European cohort of young (18-55) cryptogenic stroke	Neurology departments	581/0	**267** (excluding ASA alone)/ **304** (no atrial abnormal) + **10** ASA only	18-55; mean age 40.3	37.8± 9.7	Complete	TTE +TOE	Yes	No	Stroke	13% of t277 patients with atrial septal abnormality (PFO, ASA or both) had mRS>2 at inclusion	Aspirin 300 mg was discontinued in 20 patients because of an outcome event and in 53 for other reasons. Aspirin was replaced by another APT in 31 patients and by ACO in 18; 24 patients received no further treatment. 92% of 581 patients received APT for >90% of FU	0 for PFO + group and 1 for no PFO- group	Ischemic stroke (n=24), TIA (n=13), systemic embolism (n=1), MI (n=3)and death (n=6)	Indirect - FU with neurologists every 6 months

© 2020 American Medical Association. All rights reserved.

Paper	Recruitment period	Study type	Setting of enrolment	Cryptogenic/ Determined aetiology	Cryptogenic PFO+/ PFO-	Age cut-off; mean age	Mean (SD) FU duration (months)	Complete[&]/ incomplete/NR assessment for index event	Method of PFO diagnosis	TOAST	>24 h R test for index event?	Type of index event	Baseline mRS	Medical treatment of cryptogenic PFO+ after index event	% ICH reported	Type of recurrent event	Direct[^]/ indirect/ NR assessment for recurrent event
**Homma S, 2002***	1993-2000	Retrospective analysis of PFO in Cryptogenic Stroke Study (PICSS), nested in WARSS trial, double blind randomised trial, randomising stroke patients to either Warfarin or Aspirin. PICSS enrolled cryptogenic stroke pts in WARSS that	Unspecified	250/365	98/152	Range: 30-85; mean age 57.9±13.3	24±1,2	Not reported	TOE	Yes	Not reported	Stroke	Any disability (Glasgow score <5) in 21/98 21.4%) PFO+ and 51/152 (33.6%) PFO-	56 PFO+ patients on Aspirin 325, and 42 PFO- patients on Warfarin (INR 1.4-2.8)	Rate of major bleeds: Warfarin=1.78/100pts/y vs Asprin 1.91, rate ratio 0.93, p+1; Minor bleeds: Warfarin=22.9 vs 8/66 rate ratio 2.64.	Ischaemic stroke, Death, TIA with lesion on imaging	Unreported (but included imaging) Monthly phone call/in person FU, plus quarterly and annual in person FU.
**Homma S, 2004***		Retrospective analysis of PFO in Cryptogenic Stroke Study (PICSS), nested in WARSS trial, double blind randomised trial, randomising stroke patients to either Warfarin or Aspirin. PICSS enrolled cryptogenic stroke pts in WARSS that	Unspecified	250/365	98/ 152	Range: 30-85; mean age 57.9±13.3	24±1,2	Not reported	TOE	Yes	Not reported	Stroke	Any disability (Glasgow score <5) in 21/98 21.4%) PFO+ and 51/152 (33.6%) PFO-	56 PFO+ patients on Aspirin 325, and 42 PFO- patients on Warfarin (INR 1.4-2.8)	Not reported	Ischaemic stroke, Death, TIA with lesion on imaging	Unreported (but included imaging) Monthly phone call/in person FU, plus quarterly and annual in person FU.

© 2020 American Medical Association. All rights reserved.

Paper	Scuchlenz HW, 2005	Harrer JU, 2006	Casaubon L, 2007
Recruitment period	1992-2002	NR	1997-2003
Study type	Observational single centre Austrian cohort of cryptogenic stroke patients with PFO	Observational two-centre German cohort of cryptogenic stroke/TIA PFO/ASA patients	Observational single centre Canadian cohort of cryptogenic stroke/TIA PFO/ASA patients
Setting of enrolment	Patients referred for TOE from neurology units	Neurology departments	Emergency Departments, wards, outpatients
Cryptogenic/ Determined aetiology	280/0 (167 with PFO percutaneous closure, 113 on medical treatment only)	124/0	121/0
Cryptogenic PFO+/ PFO-	280/0	124/0 (83 patients on medical treatment only)	121/0 (61 patients on medical treatment only)
Age cut-off; mean age in cryptogenic PFO+ group	No cut-off, mean age 44±11, 50 ±12	No cut-off; mean age 51 ± 15	15-79; mean age 46
Mean (SD) FU duration (months)	Between 31.2 (25.2-37.2) for Antiplatelets and 32.4 (26.4-37.2) for Anticoagulants	50± 32	Overall median FU: 32 months
Complete[&]/ incomplete/NR assessment for index event	NR	Incomplete (no intracranial imaging)	Complete
Method of PFO diagnosis	TOE	TOE (81.5%) and TTE (28.5%) and TCD for RLS quantification	TTE and TOE to confirm shunt
TOAST	NR	NR	NR
>24 h R test for index event?	NR	No, but 90% of patients had 24h HOLTER	Yes - 48h HOLTER in selected patient
Type of index event	TIA, Stroke	Stroke (67.7%)/TIA (32.3%)	Ischaemic stroke (69%), TIA (31%)
Baseline mRS	NR	NR	NR
Medical treatment of cryptogenic PFO+ after index event	66 patients (23.6%) on Aspirin 100 mg; 47 (16.8%) on anticoagulation	59 patients (47.6%) on antiplatelets (Aspirin/ ticlopidine); 54 (43.5%) on anticoagulants (warfarin / Heparin)	34% of patients on Antiplatelets (Aspirin 81-325 mg, Clopidogrel 75 mg, Aspirin+dipiridamol); 17% on warfarin
% ICH reported	On anticoagulation, not aspirin: 1 fatal ICH, 2 GI, 2 muscle bleed, 1 [illegible]	NR	0%
Type of recurrent event in cryptogenic PFO+ [illegible]	TIA, stroke	Stroke, TIA, thrombosis, systemic or pulmonary embolism, death.	Recurrent stroke/TIA.
Direct[^]/ indirect/ NR assessment for recurrent event	Indirect	Indirect (but if recurrence suspected, imaging obtained)	Indirect (but telephone FU every 6 months and clinical review if rec reported)

© 2020 American Medical Association. All rights reserved.

Paper	Recruitment period	Study type	Setting of enrolment	Cryptogenic/ Determined aetiology	Cryptogenic PFO+/ PFO-	Age cut-off; mean age	Mean (SD) FU duration (months)	Complete[&]/ incomplete/NR assessment for index event	Method of PFO diagnosis	TOAST	>24 h R test for index event?	Type of index event	Baseline mRS	Medical treatment of cryptogenic PFO+ after index event	% ICH reported	Type of recurrent event	Direct[^]/ indirect/ NR assessment for recurrent event event
**Serena J, 2008**	200-2005	Observational, multicentre Spanish cryptogenic stroke cohort (CODICIA)	Neurology depts	486/0	297/189	≥18; mean age 56.47±13.5, 51.6±15.2	24.3±13	Complete	TCD + TTE/TOE	Yes	24 h monitoring at the most in selected cases	Stroke, TIA	NR	Of the whole population (PFO+ and PFO-), 79 % received APT, and 21%ACo (Acenocoumarol)	**no**	Stroke, TIA	Indirect (FU at 3 months and annual)
**Weimar C, 2009**	NR	Observational multicentre German cohort on crypto stroke patients who underwent either TOE or TCD	Neurology departments	1126/0	404 (351 only FU)/722 (548 only FU)	No cut-ff; mean age: 57.0	28.4	complete	TOE or TCD	Yes	NR	Stroke TIA	Mean mRS at discharge in the PFO+ medically treated group =0.9	At last follow-up: 154 AP, 63 AC and 17 none 154 (43%) APTs	no	stroke	Indirect (telephone bi-annual screening plus direct assessment if event reported)
**Lee JY, 2010**	2000-2007	Observational single centre cohort of PFO+ pts with crypto stroke	Stroke Centre	184	184/-	No cut-off; mean age 51.0± 14	Median FU 42 months	NR	TOE	yes	NR	Stroke	NR	99 patients (53.8%) on antiplatelets (Aspirin 100 in 78 pts, in monotherapy in 14 patients, or + Clopidogrel 75mg in 49 patients, or + cilostazol 100 bd in 11 patients; clopidogrel 75 mg	0%, minor bleed in Aco	Imaging-confirmed Stroke	Indirect

© 2020 American Medical Association. All rights reserved.

Paper	Recruitment period	Study type	Setting of enrolment	Cryptogenic/ Determined aetiology	Cryptogenic PFO+/ PFO-	Age cut-off; mean age	Mean (SD) FU duration (months)	Complete[&]/ incomplete/NR assessment for index event	Method of PFO diagnosis	TOAST	>24 h R test for index event?	Type of index event	Baseline mRS	Medical treatment of cryptogenic PFO+ after index event	% ICH reported	Type of recurrent event	Direct[^]/ indirect/ NR assessment for recurrent event
**Paciaroni M, 2011**	2006-2007	Observational multicentre Italian cohort of crypto TIA/minor stroke patients with PFO treated either medically or with percutaneous PFO closure	" Hospitals" (not further specified)	238/-	238/-	<55; mean age 42.2 ± 10	24	Complete	TOE	NR	NR (but 24hour ECG monitoring was routine)	74 TIA (31.1%), 164 minor stroke (68.9%)	NR (but only mRS <3 at 3 month were included)	93patients on antiplatelets (39.1%, 78 aspirin 100–325 mg, 7 ticlopidine 500 mg, 7 clopidogrel 75 mg and 1 aspirin 50 mg plus dipyridamole 400 mg) and 24 (10.1%) vitamin K antagonists.	0.8% (2 ICH/238, all in the ACo group)	Recurrent stroke; combined recurrent TIA+stroke	Indirect (neurology assessment with imaging if recurrence suspected)
**Furlan 2012, CLOSURE 1**	2003-2008	Randomised open-label trial of cryptogenic stroke/TIA patients with PFO treated either medically or with percutaneous PFO closure	Unspecified	909/-	909/-	18-60; mean age between 45.7± 9.1 and 46.3± 9.6	24	Unreported	TOE	NR	NR	Ischemic stroke (329/461), TIA (132/461	NR	252/451 patients on aspirin, 111/451 on warfarin, 40/451 on aspirin +warfarin, 38/451 on no medication.	NR	Stroke, TIA, and death.	Indirect

© 2020 American Medical Association. All rights reserved.

Paper	Recruitment period	Study type	Setting of enrolment	Cryptogenic/ Determined aetiology	Cryptogenic PFO+/ PFO-	Age cut-off; mean age	Mean (SD) FU duration (months)	Complete[&]/ incomplete/NR assessment for index event	Method of PFO diagnosis	TOAST	>24 h R test for index event?	Type of index event	Baseline mRS	Medical treatment of cryptogenic PFO+ after index event	% ICH reported	Type of recurrent event	Direct[^]/ indirect/ NR assessment for recurrent event
**Wahl A, 2012***	1994-2000	Observational single centre Swiss cohort of cryptogenic PFO+ patients from the Bern stroke registry	Bern University Hospital Stroke centre	308 cryptogenic PFO+/0	308 cryptogenic PFO+/0 (150 with PFO percutaneous closure, 158 on medical treatment, but 148 153 for follow-up	No cut-ff; mean age between 50± 12 and 50.7± 13.3	Median 132 (IQR 96-180)	Complete	TOE	NR	NR but only ECG and 24h HOLTER were routine	Ischaemic stroke, TIA	NR	158 pts on medical treatment (Warfarin or Asprin 100-300 mg) or Clopidogrel 75 mg	ICH not specified; 2 major bleeding, and 3 minor bleeding in the medical group	stroke, TIA, peripheral embolism, major bleeding, death	Indirect (but included in person assessment in case of suspected rec, with imaging)
**Horner S, 2013**	2003-2005	Cohort of cryptogenic stroke/ TIA patients with right-to-left shunt from the National Austrian multicentre registry (Austrian Paradoxical Cerebral Embolism Trial) treated either medically or with percutaneous PFO closure	15 Austrian Stroke Units	188/-	188/- patients with right-to-left shunt (pulmonary shunt included); of these, 176 cardiac right-to-left shunt (including atrial septal defect); 168 patients had PFO.	18-55; mean age 42.37± 9.69	24	Complete	TCD and TOE	NR	NR	108 (57.4%) stroke, 80 (42.5%) TIA	NR (but median NIHSS=1)	64 (81%) patients on antiplatelets, 14 (17.7%) patients on oral anticoagulants.	NR	Stroke, TIA	indirect

© 2020 American Medical Association. All rights reserved.

Paper	Recruitment period	Study type	Setting of enrolment	Cryptogenic/ Determined aetiology	Cryptogenic PFO+/ PFO-	Age cut-off; mean age	Mean (SD) FU duration (months)	Complete&/ incomplete/ NR assessment for index	Method of PFO diagnosis	TOAST	>24 h R test for index event?	Type of index event	Baseline mRS	Medical treatment of cryptogenic PFO+ after index event	% ICH reported	Type of recurrent event	Direct^/ indirect/ NR assessment for recurrent event
**Meier 2013, PC trial**	2000-2009	Randomised open-label trial of cryptogenic stroke/TIA patients with PFO treated either medically or with percutaneous PFO closure	NR	414/-	414/-	<60; mean age 44.3-44.6	48	Complete	TOE	NR	NR	Ischemic stroke (163/210)/ TIA (42/210); peripheral embolism (0/210)	NR	Aspirin 139/206; thienopyridine 20/206; AC 61/206	NR	Composite of death, non fatal stroke, TIA and peripheral embolism	Indirect (Appendix)
**Moon J, 2016**	2010-2014	Observational single centre Korean cohort of cryptogenic stroke/TIA patients with PFO treated either medically or with percutaneous PFO closure	NR	164/-	164/-	18-60; mean age 48.1 ± 8.5	20	NR	TOE	NR	NR	Recurrent ischaemic stroke (n=1), TIA (n=0), death (n=1), peripheral embolism (n=0)	NR	78/92 patients (56%) on antiplatelets, 69 on Aspirin, 44 on Clopidogrel, 6/92 on anticoagulants	0%	Composite of death, isch stroke, TIA, peripheral embolism	Unreported

© 2020 American Medical Association. All rights reserved.

Paper	Recruitment period	Study type	Setting of enrolment	Cryptogenic/ Determined aetiology	Cryptogenic PFO+/ PFO-	Age cut-off; mean age	Mean (SD) FU duration (months)	Complete[&]/ incomplete/ NR assessment for index	Method of PFO diagnosis	TOAST	>24 h R test for index event?	Type of index event	Baseline mRS	Medical treatment of cryptogenic PFO+ after index event	% ICH reported	Type of recurrent event	Direct[^]/ indirect/ NR assessment for recurrent event
**Danese A, 2017***	2006-2015	Observational single centre Italian cohort of Cryptogenic stroke/TIA patients with PFO treated either medically or with percutaneous PFO closure	Neurology/Medicine/Stroke Unit admissions	159/150	159/0	<55; mean 44.5 ±9.7	51.6±34.8	Complete	TCD and TOE	Yes	No	Stroke, TIA	NR	Aspririn 100 mg, Ticlopidine 250 mg bd, Clopidogrel 75 mg, Warfarin	0%	Stroke, TIA	Direct
**Mas 2017,CLOSE**	2008-2016	Randomised open-label trial of cryptogenic stroke/TIA patients with PFO treated either medically or with percutaneous PFO closure	NR	663/-	663/-	16-60; mean age 43.8± 10	62.4 ± 25.2	Complete	TOE	NR	501/596 patients had monitoring for several days	Imaging-proved stroke independently from clinical duration	mRS 0-1: 189/235 patients	In the antiplatelet-only group and the PFO closure group: 410 patients (86.7%) received aspirin, 51 (10.8%) clopidogrel, 6 (1.3%) aspirin dipyridamole, 6 (1.3%) aspirin + clopidogrel. (Not significantly different between the PFO closure group and the antiplatelet-only group)	0%	Fatal/non fatal stroke	Indirect

© 2020 American Medical Association. All rights reserved.

Paper	Recruitment period	Study type	Setting of enrolment	Cryptogenic/ Determined aetiology	Cryptogenic PFO+/ PFO-	Age cut-off; mean age	Mean (SD) FU duration (months)	Complete&/ incomplete/ NR assessment for index	Method of PFO diagnosis	TOAST	>24 h R test for index event?	Type of index event	Baseline mRS	Medical treatment of cryptogenic PFO+ after index event	% ICH reported	Type of recurrent event	Direct^/ indirect/ NR assessment for recurrent event
**Saver 2017, RESPECT***	2003-2011	Randomised open-label trial of cryptogenic stroke/TIA patients with PFO treated either medically or with percutaneous PFO closure	NR	980/-	980/-	18-60; mean age 46.2	Median FU: 70.8 months	Complete	TOE	yes	NR	Ischaemic stroke / neuroimaging positive TIA	NR	Of 481 patients on medical treatment, Aspirin (% FU time: 54%), Warfarin (19%), Clopidogrel (12%), Aspirin +Dipyridamole (8%), Aspirin+Clopidogrel (3%), other (3%) and none (2%)	1 ICH/ 481	Recurrent nonfatal ischaemic stroke, fatal ischaemic stroke or early death	Indirect but included imaging in case of suspected event.
**Søndegaard 2017, GORE REDUCE**	2008-2015	Randomised open-label trial of cryptogenic stroke/TIA patients with PFO treated either medically or with percutaneous PFO closure	NR	664/-	664/-	18-59; mean age 45.2	Median 38.4 (IQ 26.4-57.6)	Complete	TOE	No	NR	199 Ischaemic stroke and 24 neuroimaging positive TIA	NR	223 patients on medical therapy (antiplatelet only permitted); % last reported: aspirin 59%, Dipyridamole 0.5%, Aspirin+Dipyridamole 6.3%, Clopidogrel 25.9%, other 4.3%, missing 4.1%	NR	Co-primary end-point: stroke and new silent infarction (on imaging only) .	Indirect but included imaging in case of suspected event.

© 2020 American Medical Association. All rights reserved.

Paper,	Recruitment period	Study type	Setting of enrolment	Cryptogenic/ Determined aetiology	Cryptogenic PFO+/ PFO-	Age cut-off; mean age	Mean (SD) FU duration (months)	Complete[&]/ incomplete/ NR assessment for index	Method of PFO diagnosis	TOAST	>24 h R test for index event?	Type of index event	Baseline mRS	Medical treatment of cryptogenic PFO+ after index event	% ICH reported	Type of recurrent event	Direct[^]/ indirect/ NR assessment for recurrent event
**Kasner 2018, NAVIGATE-ESUS**	2014-2017	Randomised double-blinded trial on efficacy and safety of rivaroxaban versus aspirin for secondary stroke prevention after ESUS	NR	7209/0	534/6675 (but PFO- group included " not reported" , with potential underestimation of PFO+ group)	>50, PFO+ mean age 64.6 ±9.2; PFO- 67.1± 9.8	17.76	Complete	TTE and/ or TOE	No (ESUS International Working Group classification)	ECG monitoring >=48h in 2433/7209 patients)	ESUS	mRS 0-2 in 498/534 patients with PFO	259/534 on Rivaroxaban 15 mg ; 275/534 on Aspirin 100 mg	NR	Stroke and peripheral embolism	Indirect
**Lee 2018, DEFENSE-PFO**	2011-2017	Randomised open-label trial on cryptogenic stroke with high risk PFO (ASA, septum hypermobility or PFO>2 mm during Valsalva)	NR	120/-	120/-	No cut-off; mean: 51.8	Median FU 33.6 (IQR: 10.8-50.4)	Complete	TOE	NR	NR	Imaging-proved stroke independently from clinical duration	mRS 0-1: 45/60	Aspirin, Aspirin+Clopi, Aspirin + cilostazol, Warfarin<br><br>At 30 days: 10/60 single antiplatelet, 35/60 dual antiplatelet and 15/60 warfarin	1/53 on Warfarin, and 1/53 haemorrhagic transformation while on dual APT (Per protocol analysis)	Composite stroke, vascular death and major bleed;<br><br>Secondary outcome: silent brain infarctions	NR

© 2020 American Medical Association. All rights reserved.

Paper	Recruitment period	Study type	Setting of enrolment	Cryptogenic/ Determined aetiology	Cryptogenic PFO+/ PFO-	Age cut-off; mean age	Mean (SD) FU duration (months)	Complete[&]/ incomplete/NR assessment for index event	Method of PFO diagnosis	TOAST	>24 h R test for index event?	Type of index event	Baseline mRS	Medical treatment of cryptogenic PFO+ after index event	% ICH reported	Type of recurrent event	Direct[^]/ indirect/ NR assessment for recurrent event
**Nezu T, 2018**	2008-2012	Observational Japanese cohort of patients with cryptogenic stroke	Kawasaki Medical School Hospital	295 (266 with follow-up) /1043	62/204	No cut-off, mean age 71.6	Median FU 1093 days, 91 months	NR	Ce-TCD and /or TOE	yes	NR	Ischaemic stroke, haemorrhagic stroke, stroke of unknown type, death	Median (interquartile range) mRS at discharge in the PFO+ group = 3 (1-4)	205 pts on APTs, 19 on ACo	7 pts with ICH	Stroke (including haemorrhagic), and all-cause mortality	Indirect
**Diener 2019, RESPECT ESUS**	2014-2018	International, double blind, parallel-group, randomized trial of Dabigatran vs Aspirin in secondary stroke prevention	NR	5390/-	680/-	≥18 (depending on time from index stroke and vascular risks factors); mean age 64.2	Median FU19 months (IQR: 13-27	Complete	NR	No (ESUS International Working Group classification)	ECG monitoring >20 h in 14% of patients, and in 6% implantable loop recorder	ESUS	Median mRS:1 (IQR=0-2)	319/680 patients on Dabigatran (150 mg twice daily, or 110 mg twice daily if age≥ 75 or estimated creatinine clearance 30 - 50 ml/min); 361/680 on Aspirin 100 mg	NR for PFO subgroup	Recurrent stroke of ischemic, hemorrhagic, or unspecified type	Indirect

© 2020 American Medical Association. All rights reserved.

Paper	Recruitment period	Study type	Setting of enrolment	Cryptogenic/ Determined aetiology	Cryptogenic PFO+/ PFO-	Age cut-off; mean age	Mean (SD) FU duration (months)	Complete[&]/ incomplete/NR assessment for index event	Method of PFO diagnosis	TOAST	>24 h R test for index event?	Type of index event	Baseline mRS	Medical treatment of cryptogenic PFO+ after index event	% ICH reported	Type of recurrent event	Direct[^]/ indirect/ NR assessment for recurrent event
**OXVASC 2019**	01/09/ 2014 and 31/03/2019	Population-based study	TIA/stroke emergency clinic based at the John Radcliffe Hospital, Oxford, UK	416/380	153/263	≥18; mean age 66.7±13.7	34 (16) months for cryptogenic TIA/stroke	Complete	Bubble TCD	yes	yes	Non-disabling stroke, TIA	mRS≤2	409/416 on antiplatelet treatment alone; 3 on anticoagulants and 4 on both antiplatelet treatment and anticoagulants.	Ischaemic event only are screened fro PFO	Ischaemic stroke	Direct

© 2020 American Medical Association. All rights reserved.

eTable 2: Quality assessment for included studies with the Newcastle-Ottawa Scale.

Paper	Year of publication	Selection	Comparability	Outcome/exposure	Quality
Mas	1995	4	2	2	good
Mas	2001	4	2	3	good
Homma	2004	4	2	3	good
Schuchlenz	2005	2	2	3	fair
Harrer	2006	4	1	3	good
Casaubon	2007	4	2	3	good
Serena	2008	4	2	3	good
Weimar	2009	4	2	2	good
Lee	2010	3	1	3	good
Paciaroni	2011	4	2	3	good
Furlan	2012	4	2	3	good
Wahl	2012	4	2	2	good
Horner	2013	4	0	3	poor
Meier	2013	4	2	3	good
Moon	2016	3	0	2	poor
Mas	2017	4	2	3	good
Saver	2017	4	2	3	good
Søndegaard	2017	4	2	3	good
Danese	2017	4	0	3	poor
Kasner	2018	4	2	3	good
Lee	2018	4	0	2	poor
Nezu	2018	4	2	2	good
Diener	2019	4	2	3	good
OXVASC	2019	4	1	3	good

© 2020 American Medical Association. All rights reserved.

eFigure 2. Sub-group analysis on the absolute risk of recurrent ischaemic stroke calculated on the 23 studies reporting this outcome. * Randomised trials.

Study	Mean age (years)	Events / Patient-Years	Risk (per 100 patient-years)	95% CI
Mas JL, 1995	39.4	2 / 203.3	0.98	0.25-3.93
Mas JL, 2001	40.3	12 / 841.05	1.43	0.81-2.51
Homma, 2004*	55.1	10 / 196	5.10	2.75-9.48
Scuchlenz HW, 2005	47.7	8 / 298.32	2.68	1.34-5.36
Harrer JU, 2006	52.5	6 / 346.11	1.73	0.78-3.86
Casaubon L, 2007	50.3	6 / 164.7	3.64	1.64-8.11
Serena J, 2008	53.2	16 / 594	2.69	1.65-4.40
Weimar C, 2009	57	20 / 554.58	3.61	2.33-5.59
Lee JY, 2010	53	14 / 556.5	2.52	1.49-4.25
Paciaroni M, 2011	40.9	6 / 234	2.56	1.15-5.71
Furlan AJ, 2012*	45.7	13 / 750	1.73	1.01-2.99
Wahl A, 2012	50.7	15 / 1683	0.89	0.54-1.48
Horner S, 2013	43.7	3 / 158	1.90	0.61-5.89
Meler B, 2013*	44.6	5 / 840	0.60	0.25-1.43
Moon J, 2016	50.2	1 / 153.64	0.65	0.09-4.62
Danese A, 2017	44.5	2 / 352	0.57	0.14-2.27
Mas JL, 2017*	43.8	14 / 1222	1.15	0.68-1.93
Saver JL, 2017*	46.2	28 / 2837.9	0.99	0.68-1.43
Sondegaard L, 2017*	44.8	12 / 713.6	1.68	0.96-2.96
Kasner SE, 2018*	64.6	20 / 491.58	4.07	2.63-6.31
Lee PH, 2018*	54	5 / 168	2.98	1.24-7.15
Nezu T, 2018	73.1	6 / 186	3.23	1.45-7.18
OXVASC, 2019	66.7	9 / 440	2.05	1.06-3.93
TOTAL		233 / 13984.3	1.94	1.51-2.50

Heterogeneity p<0.0001

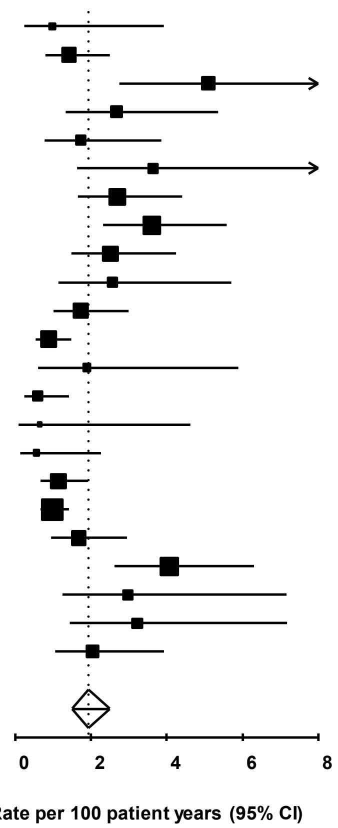

© 2020 American Medical Association. All rights reserved.

e-Table 3: Sample size calculation for for trials of PFO closure versus medical treatment only after cryptogenic TIA/stroke in patients ≥ 60 years of age, with 80% power to detect either a 66% or a 33% risk reduction for the interventional closure arm, assuming a risk of recurrent ischaemic stroke of 2/100-patient-years in the medical arm. FU= Follow-Up

Hypothetical Risk Reduction	Medical Arm	Interventional Arm	Sample size
66%	Risk per 100-person-year=2; over 5 years of FU= 10%	Risk per 100-person-year = 0.66; over 5 years of FU= 3.3%	432 (216 per arm)
33%		Risk per 100-person-year=1.33; over 5 years of FU= 6.67 %	2160 (1080 per arm)

© 2020 American Medical Association. All rights reserved.
